# Supplementary material for: Conformational plasticity of ligand-bound and ternary GPCR complexes studied by 19F NMR of the β1-adrenergic receptor
Source: Nat Commun. 2020 Feb 3;11:669. doi: 10.1038/s41467-020-14526-3 (PMC6997182; doi:10.1038/s41467-020-14526-3)
Supplement: Supplementary file 1 — Supplementary Information [file 41467_2020_14526_MOESM1_ESM.pdf]

Supplementary Information

Conformational plasticity of ligand-bound and ternary GPCR complexes studied by ^{19}F NMR of the β_1-adrenergic receptor

J. Niclas Frei et al.

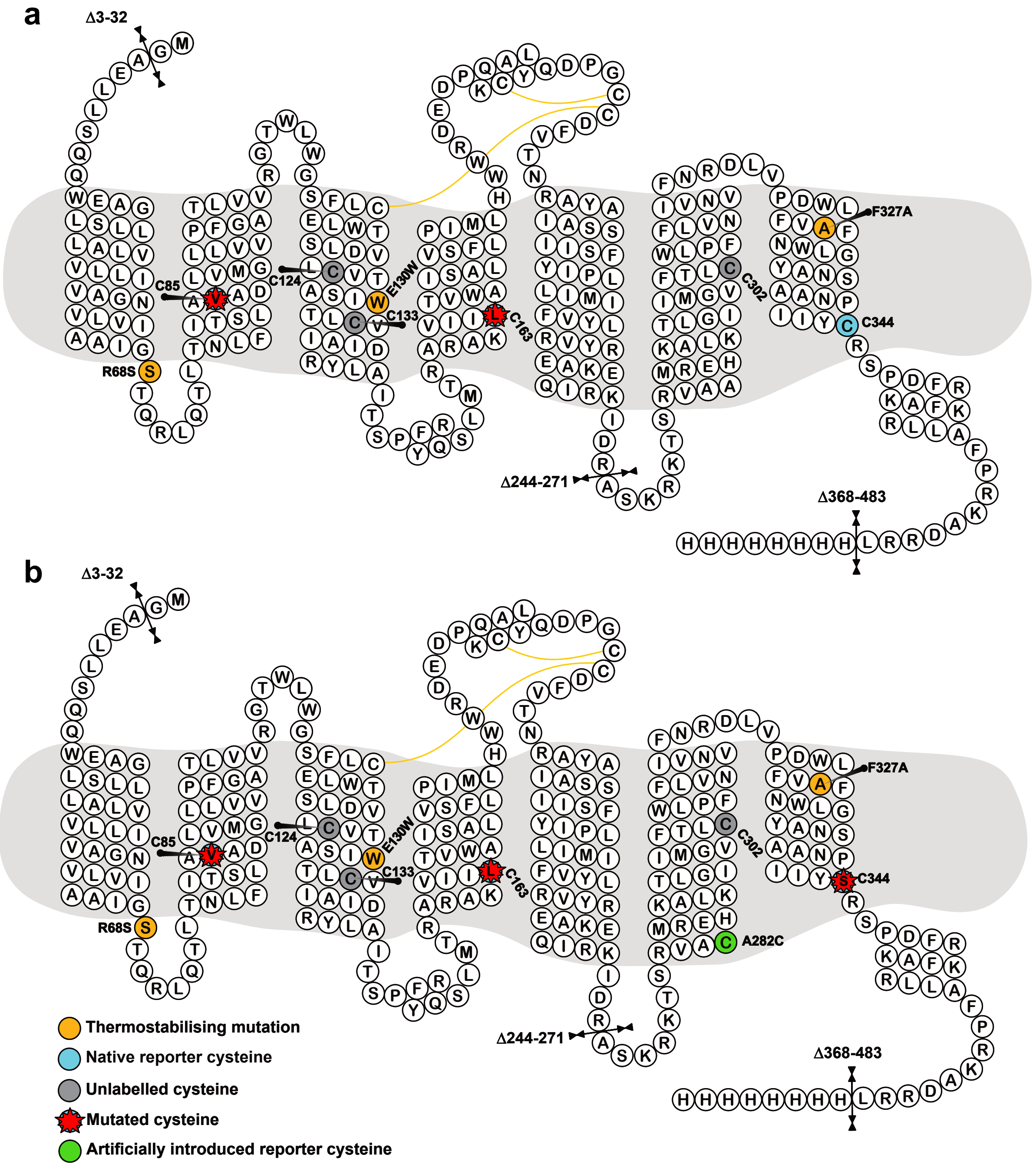

Supplementary Fig. 1 Snake diagrams of the β_1AR constructs used for ^{19}F NMR studies. (a) The construct β_1AR-m-CysΔ2 used to study ^{TET}C344$^{7.54}$ contains three thermostabilising mutations R68S, E130W and F327A (orange circles). The reporter C344$^{7.54}$ to be labelled with TET is shown in cyan and necessary cysteine deletion mutations C85V$^{2.48}$ and C163L$^{4.47}$ are shown as red stars. Cysteines inert to TET labelling under the given reaction conditions are shown in grey. **(b)** The construct β_1AR-m-TM6-CysΔ2 used to study A282C$^{TET,\ 6.27}$, with the residue colour coding identical to (a). In addition, the native reporter cysteine C344$^{7.54}$ was mutated to C344S while the reporter cysteine A282C (green circle) serving as TET labelling site was introduced.

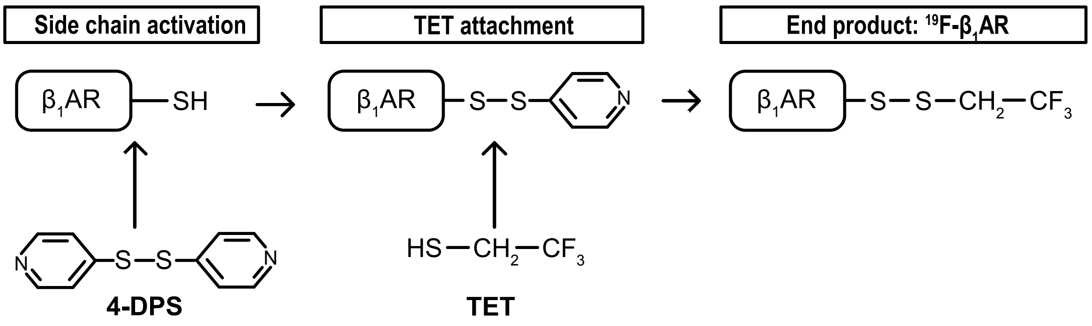

Supplementary Fig. 2 ^{19}F labelling of a cysteine sulfhydryl group of β_1AR with TET. In a first step, accessible cysteine side chains of the β_1AR are activated with 4,4'-Dipyridyl disulphide (4-DPS). Subsequently, the activated side chain is treated with 2,2,2'-Trifluoroethanethiol to produce the ^{19}F labelled β_1AR [1].

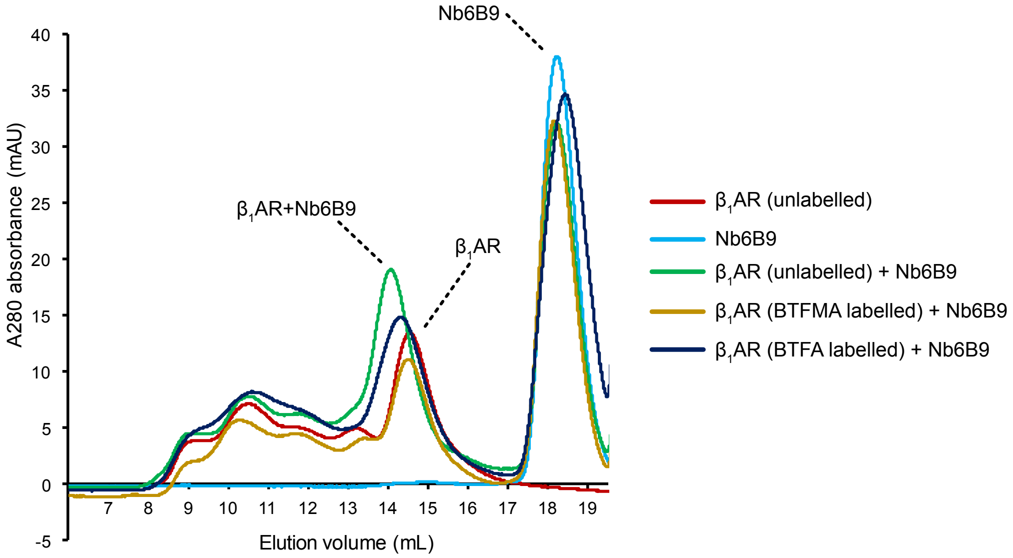

Supplementary Fig. 3 Analytical size exclusion chromatography of β_1AR tagged with various ^{19}F reagents. Size exclusion Superdex S200 10/300 chromatography traces are shown for untagged β_1AR (red), free Nb6B9 (cyan), untagged β_1AR in the presence of Nb6B9 (green), β_1AR tagged with BTFMA in the presence of Nb6B9 (orange) and β_1AR tagged with BTFA in the presence of Nb6B9 (purple). All samples were treated with 1 mM isoprenaline prior to the addition of Nb6B9. Peak positions for the individual components are indicated by labels with dotted lines. Binding of the Nb6B9 to functional β_1AR is indicated by a left shift in the elution volume from 14.4 mL for free receptor to 14.1 mL for the ternary complex.

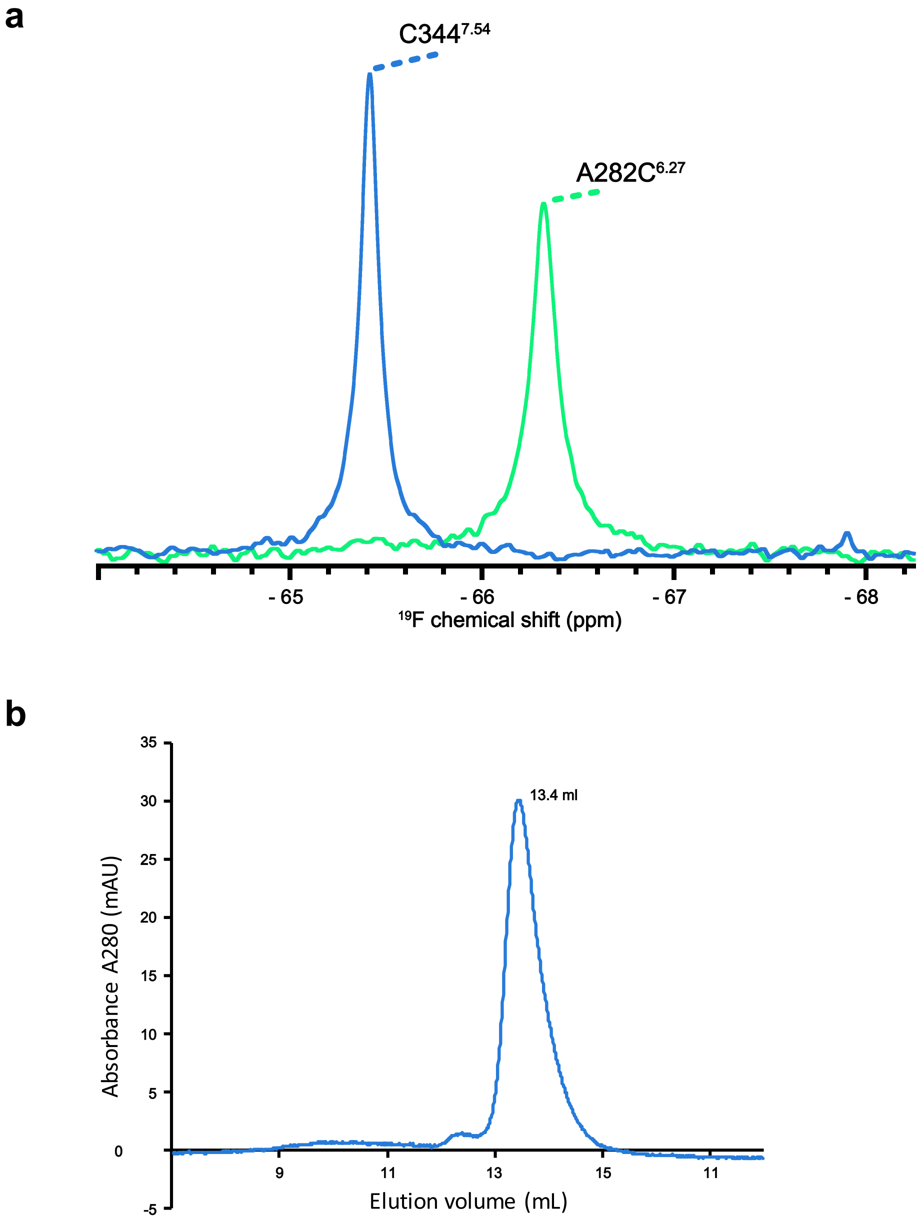

Supplementary Fig. 4 β_1AR ^{19}F NMR samples. (a) Assignment of ^{TET}C344$^{7.54}$ and A282C$^{TET,\ 6.27}$ signals. Individual ^{19}F NMR spectra of β_1AR in the apo form were recorded at 564 MHz for ^{TET}C344$^{7.54}$ (blue) using constructs β_1AR-m-CysΔ2, and for A282C$^{TET,\ 6.27}$ (light green) using construct β_1AR-m-TM6-CysΔ2, respectively. With the exception of the positions 344 and 282, the two constructs are identical. The observation of a single peak for ^{TET}C344$^{7.54}$ and for A282C$^{TET,\ 6.27}$, respectively, allowed therefore the unambiguous assignment of the two labelling positions as C344 and A282C. The absence of additional background signals and the non-overlapping nature of the two NMR signals shows that no additional cysteine residues were ^{19}F labelled. All spectra were obtained at 308 K and were scaled to match their signal integrals. **(b)** Size exclusion Superdex S200 10/300 chromatography trace of apo β_1AR-m-CysΔ2 ^{TET}C344$^{7.54}$ following purification by alprenolol affinity chromatography. The high level of receptor purity and homogeneity displayed is representative of all the NMR samples used in this study.

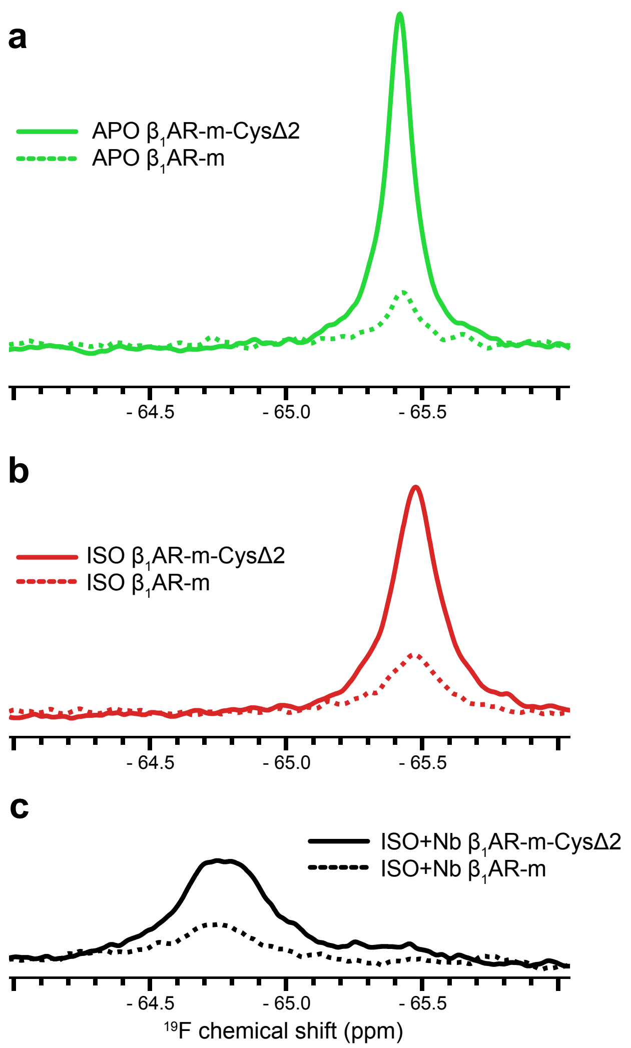

Supplementary Fig. 5 Comparison of ^{19}F NMR spectra following TET labelling of the β_1AR-m and β_1AR-m-CysΔ2 constructs. ^{19}F NMR spectra (564 MHz) were recorded under identical conditions at 308 K for the apo receptor (green) **(a)**, receptor bound to the full agonist isoprenaline (red) **(b)**, and in ternary complex between the β_1AR, full agonist isoprenaline and the G_s mimicking nanobody Nb6B9 (black) **(c)**. Spectra of β_1AR-m-CysΔ2 are shown as solid lines and of β_1AR-m as dashed lines, respectively. Corresponding spectra of both constructs are virtually identical, except for the lower intensity for β_1AR-m, which is evidence for the reduced yield in protein uniquely labelled at ^{TET}C344$^{7.54}$ when using this construct. Labelling of β_1AR-m at multiple Cys sites that compete with C344 lead to insoluble protein that was easily separated from the wanted ^{TET}C344$^{7.54}$ product, but resulted in dramatically reduced yields, which in view of their identical spectroscopic behaviour justified the use of the β_1AR-m-CysΔ2 for ^{19}F NMR spectroscopy.

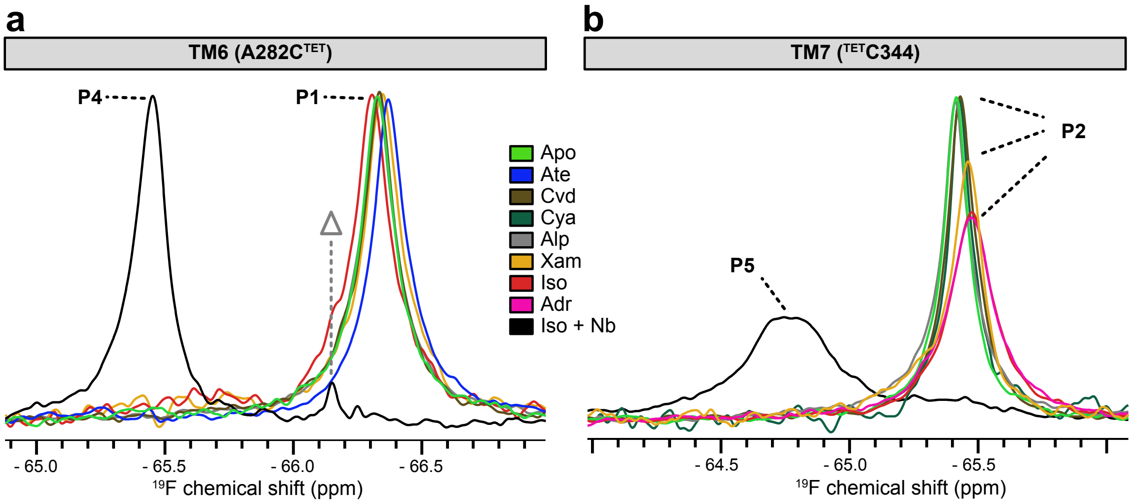

Supplementary Fig. 6 ^{19}F NMR spectra of TM6 ^{TET}C344$^{7.54}$ and TM7 A282C$^{TET, 6.27}$ for ligand bound states of β_1AR investigated in this study. ^{19}F NMR spectra (564 MHz) are shown as overlays for all ligands and the ternary complex with full agonist isoprenaline and nanobody Nb6B9 for TM6 A282C$^{TET, 6.27}$ **(a)** and TM7 ^{TET}C344$^{7.54}$ **(b)**. The colours for the individual ligands bound are indicated in the legend on the figure. All spectra were obtained at 308 K and are shown scaled according to their signal integrals. The positions for peaks P1, P2, P4 and P5 representing functional states of β_1AR as discussed in the main text are indicated.The signal marked with Δ relates to the release of TET due to slow cleavage of the S-S bond at 308 K. Values for the chemical shift positions and linewidths of the signals can be found in Supplementary Table 2.

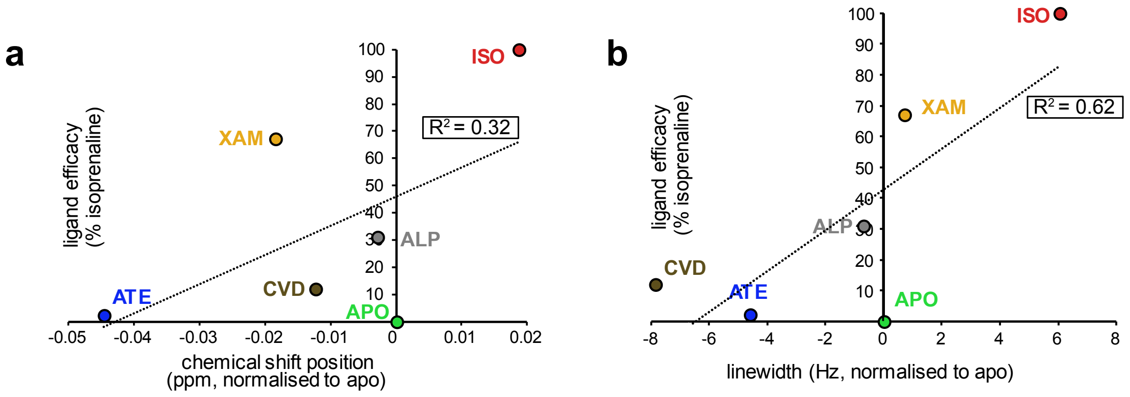

Supplementary Fig. 7 TM6 A282C$^{TET,6.27}$ does not show any significant linear correlation beween the P1 ^{19}F NMR chemical shift and ligand efficacy, or between the P1 linewidth and ligand efficacy. (a) The correlation between the chemical shift position of P1 and the efficacy of ligands towards G_s signalling shows a very weak correlation. The following conditions were assessed: apo receptor (green), atenolol (blue), carvedilol (brown), alprenolol (grey), xamoterol (orange) and isoprenaline (red). All shift positions were normalised to the chemical shift of P1 for the apo receptor at −66.33 ppm. G_s efficacies were normalised to the isoprenaline G_s efficacy which was set to 100%. A dotted line shows the best linear fit of the data points and an R^2 value of 0.32 indicates a relatively poor fit. **(b)** Similarly, the correlation of the linewidth of P1 with the G_s efficacy is shown using the same receptor states as in (a). Linewidths were measured as widths of P1 at the peak half-height. All linewidths were normalised relative to the linewidth of P1 for the apo receptor (73 Hz). G_s efficacies are normalised to the isoprenaline G_s efficacy which was set to 100%. The relatively poor quality of the linear fit is indicated by an R^2 value of 0.62.

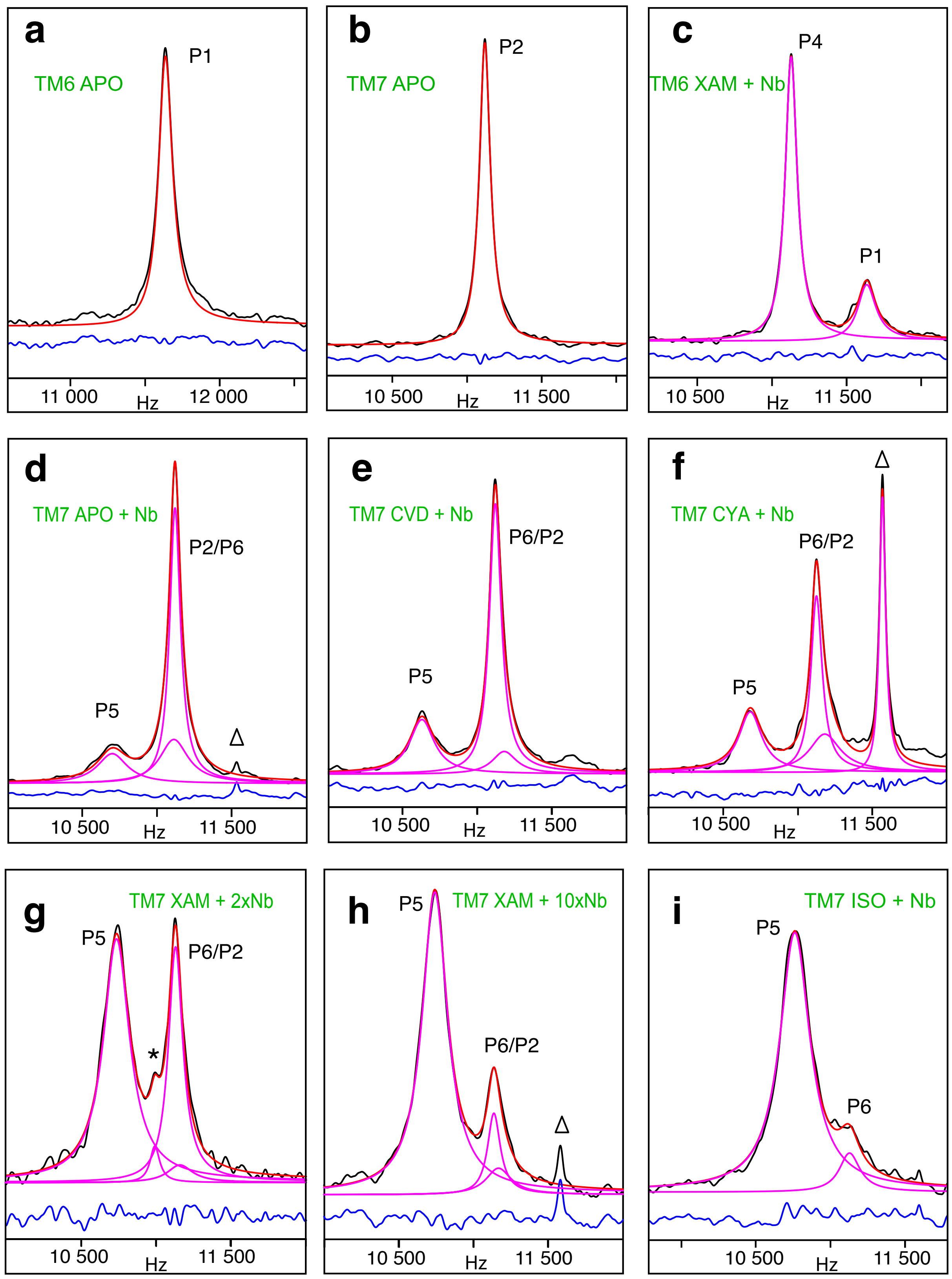

Supplementary Fig. 8 1D ^{19}F spectrum deconvolutions. Overlay of 1D ^{19}F NMR spectra (experimental data) (black), the spectral deconvolutions as Lorentzian signals (purple), the simulated 1D spectra consisting of the sum of the individual deconvoluted signals (red), and the residual error of the deconvolutions (blue). Each spectrum corresponds to a region of 2,000 Hz centered on the observable peaks. The peak labelled Δ corresponds to free TET that is released

over time due to slow cleavage of the S-S bond at 308 K. The signal labelled with * is a degradation product of unknown composition (R_2 = 60 Hz). β_1AR TM6, A282C$^{TET, 6.27}$: **(a)** apo form, **(c)** apo receptor in the presence of Nb6B9. β_1AR TM7, ^{TET}C344$^{7.54}$: **(b)** apo form, **(d)** apo receptor in the presence of Nb6B9, **(e-i)** agonist bound receptor in the presence of Nb6B9, with carvedilol **(e)**, cyanopindolol **(f)**, xamoterol **(g,h)**, and isoprenaline **(i)**. Excess ratio of nanobody over receptor was two-fold except for (h) were the excess was ten-fold. All the apo and ligand bound receptor spectra for TM6 (A282C$^{TET, 6.27}$) and TM7 (^{TET}C344$^{7.54}$) (see Supplementary Table 2) were deconvoluted with a single Lorentzian line for P1 (a) and for P2 (b), respectively. The downfield signal for the spectra in the presence of nanobody was deconvoluted as a single line both for TM6 P4 (c) and for TM7 P5 (d-i), respectively. The upfield signal was deconvoluted as a single signal for TM6 P1 (c) (see Supplementary Table 2), two signals for the P2/P6 region of TM7 (d-h), and as a single signal for the P2/P6 region of the isoprenaline bound ternary receptor TM7 (i).

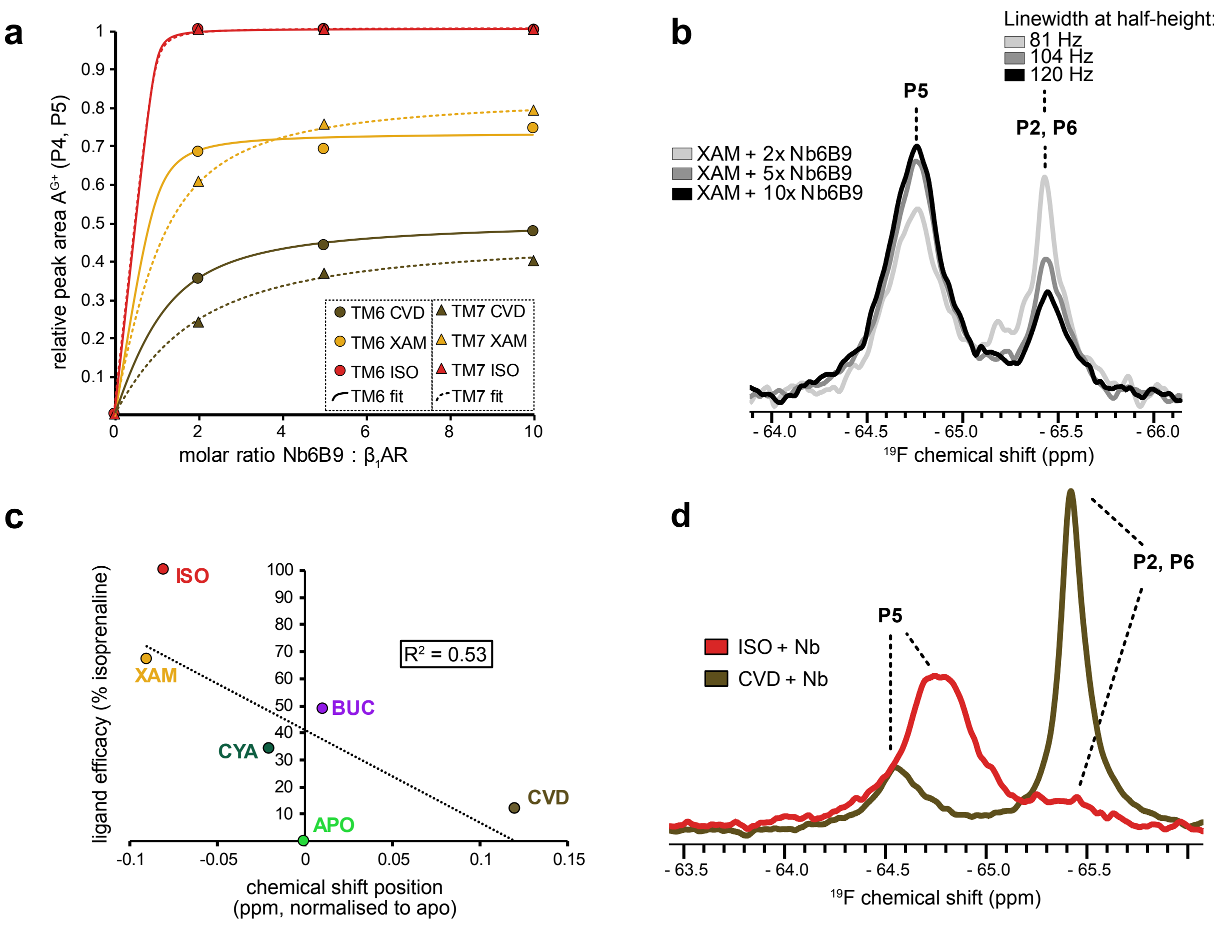

Supplementary Fig. 9 ^{19}F NMR analysis of ternary complex formation.

(a) Nanobody Nb6B9 was titrated to the receptor in molar nanobody excesses of twofold, fivefold and tenfold with the receptor individually labelled at positions $^{TET}C344^{7.54}$ or $A282C^{TET, 6.27}$. The amount of active ternary receptor (A^{G+}) formed was monitored as the peak integral of P4 for $A282C^{TET, 6.27}$ or P5 for $^{TET}C344^{7.54}$, respectively. The amounts of P4 and P5 reach a plateau as the amount of nanobody is increased. Circles indicate the relative peak area of P4 for $A282C^{TET, 6.27}$ and triangles show the relative peak area of P5 for $^{TET}C344^{7.54}$. Data was collected for ternary complexes with ligands carvedilol (brown), xamoterol (orange) and isoprenaline (red). **(b)** Overlay of individual 1D ^{19}F NMR spectra of the nanobody titration leading to the formation of the ternary complex P5 (A^{G+}) in increasing amounts as measured by $^{TET}C344^{7.54}$, using a twofold (light grey), fivefold (dark grey) and tenfold (black) nanobody molar excess. The positions of P5 (A^{G+}) and the overlapped region consisting of P2 ($I_{1,2}$) and P6 (A^{G-}) are indicated by dotted lines. With increasing excess of nanobody, the linewidth of the broader peak P6 increasingly dominates the overall linewidth of the overlapped P2/P6 region. The latter agrees with an increase in the amount of the broader P6 (A^{G-}) with the amount of the sharper P2 ($I_{1,2}$) decreasing as nanobody is added in larger excess. The linewidths of this overlapped P2/P6 region are given above the peak annotation. **(c)** Correlating the chemical shift positions of the P5 signal of $^{TET}C344^{7.54}$ for the active nanobody-

bound complexes (A^{G+}) with the ligand efficacy reveals a poor linear fit ($R^2 = 0.51$). Complexes (A^{G+}) were formed for the apo receptor (light green) and with β_1AR bound to the ligands carvedilol (brown), cyanopindolol (dark green), bucindolol (purple), xamoterol (orange) and isoprenaline (red), respectively. **(d)** Differences in the chemical shift positions of the P5 signal for ternary complexes (A^{G+}) with the receptor bound to different agonists are revealed. The ^{19}F NMR spectra of ^{TET}C344$^{7.54}$ for the ternary complexes with ligands isoprenaline (red) and carvedilol (brown) were recorded in the presence of a twofold molar excess of nanobody. The positions of the peaks are indicated by dotted lines. Individual chemical shift and linewidth informations are given in Supplementary Table 2.

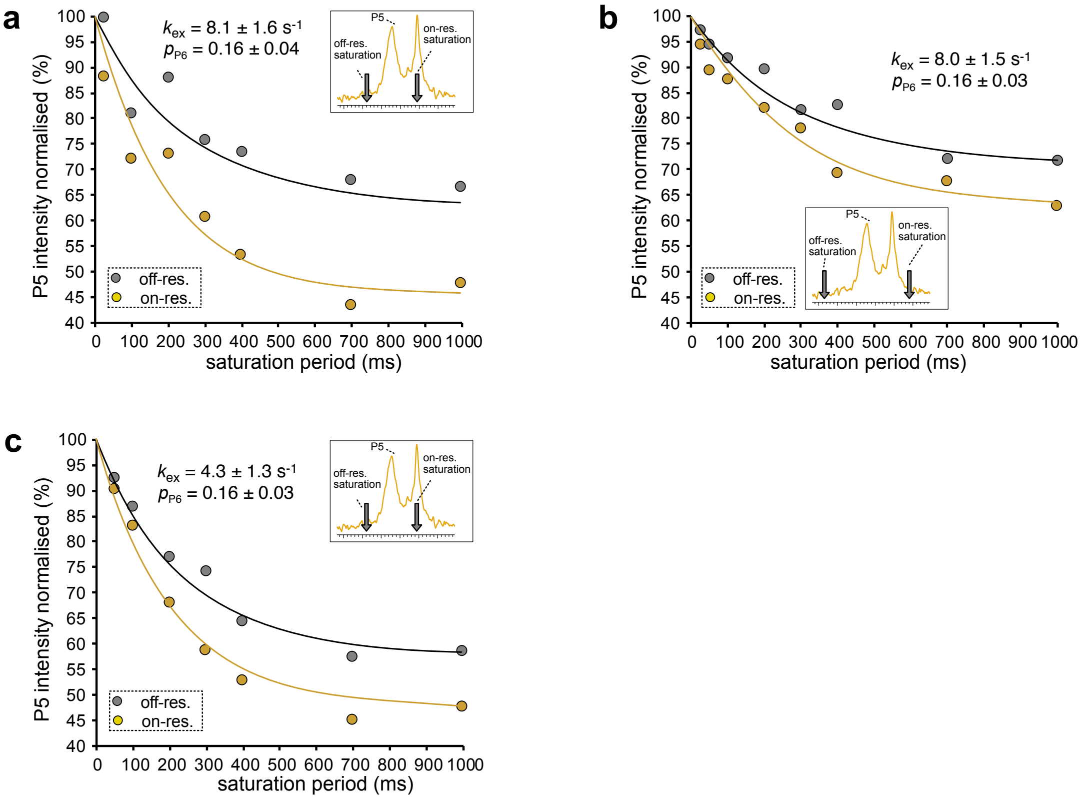

Supplementary Fig. 10 ^{19}F NMR saturation transfer time courses for β_1AR in ternary complex with xamoterol and Nb6B9. The plots show normalised signal intensities for P5 following on- and off-resonance saturation. The insets show the positions of the saturation field (grey arrows) relative to the observed peak P5 (A^{G+}). **(a)** The saturation field (field strength 25 Hz) was positioned 384 Hz upfield from P5 (A^{G+}) and the intensities of the P5 signal measured for saturation times ranging from 25 ms to 1000 ms (yellow dots). A second off-resonance reference experiment was conducted with the saturation field positioned 384 Hz downfield from P5 (grey dots). The on-resonance position corresponds to the location of the broad peak P6 (A^{G-}). **(b)** Similar saturation transfer time course with the saturation field positioned 684 Hz upfield (yellow dots) and downfield (grey dots) from P5, to selectively saturate the broad foot of the upfield P6 peak, while minimizing saturation of P2. The exchange rates k_{ex} from (a) and (b) obtained from simultaneously fitting on-resonance and off-resonance (reference) experiments are very similar, suggesting that the exchange is taking place between P5 and P6, without any involvement of P2. **(c)** Similar time course to (a) but using a ten-fold excess in Nb6B9, while a two-fold excess was used for (a) and (b). The data shows the normalised intensities of P5 after saturation at the respective positions shown in the insets displaying the spectra. Compared to the saturation in (a) the response in (b) is reduced.

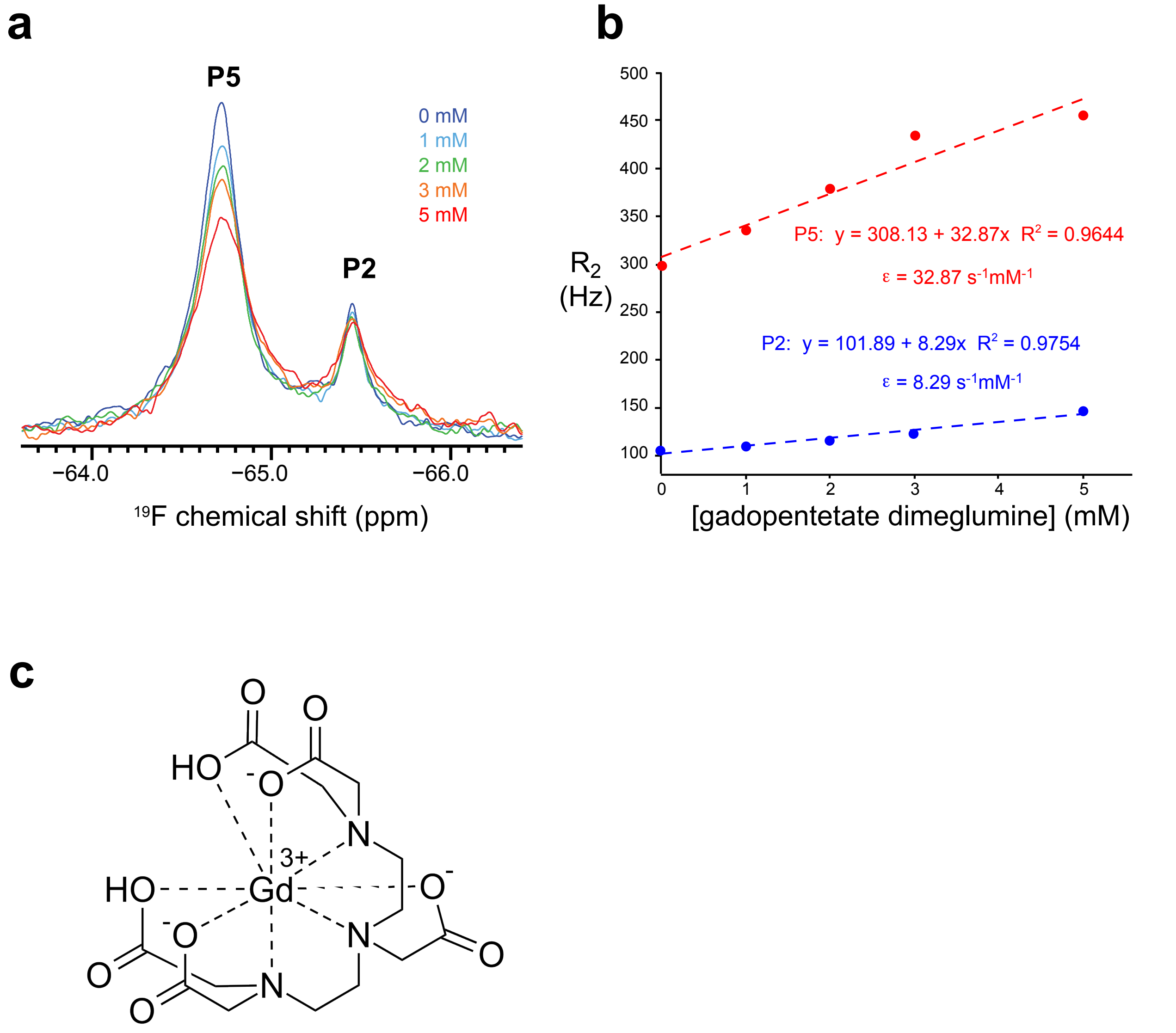

Supplementary Fig. 11 Probing the solvent-accessibility of TM7 ^{TET}C344$^{7.54}$ through the addition of the Gd^{3+} paramagnetic relaxation reagent gadopentetate dimeglumine (Magnevist). (a) Intensities of the P5 signal of the ternary xamoterol-bound receptor coupled to nanobody Nb6N9 and the P2 signal of xamoterol-bound β_1AR following addition of increasing amounts of Magnevist (0 mM (blue), 1 mM (light blue), 2 mM (green), 3 mM (orange), 5 mM (red)). Under the chosen starting conditions (1 mM xamoterol, two-fold Nb6B9 excess) the initial ratio (0 mM Gd^{3+}) of P5 to P2 was 60:40, allowing a side-by-side assessment of the effect of Gd^{3+} addition on P2 and P5. (b) R_2 rates for P5 (red) and P2 (blue) determined from a two-point relaxation measurement (Intensity in a CPMG experiment with ν_{CPMG} = 5,000 Hz and a constant time of 2.5 ms versus a CPMG reference plane without the 2.5 ms delay). The relaxation enhancements ε due to the addition of the relaxation reagent are given by the slopes of the linear fits, as 32.87 $s^{-1}mM^{-1}$ for the ternary complex and 8.29 $s^{-1}mM^{-1}$ for the ligand-bound state. **(c)** Molecular structure of gadopentetate dimeglumine (Magnevist).

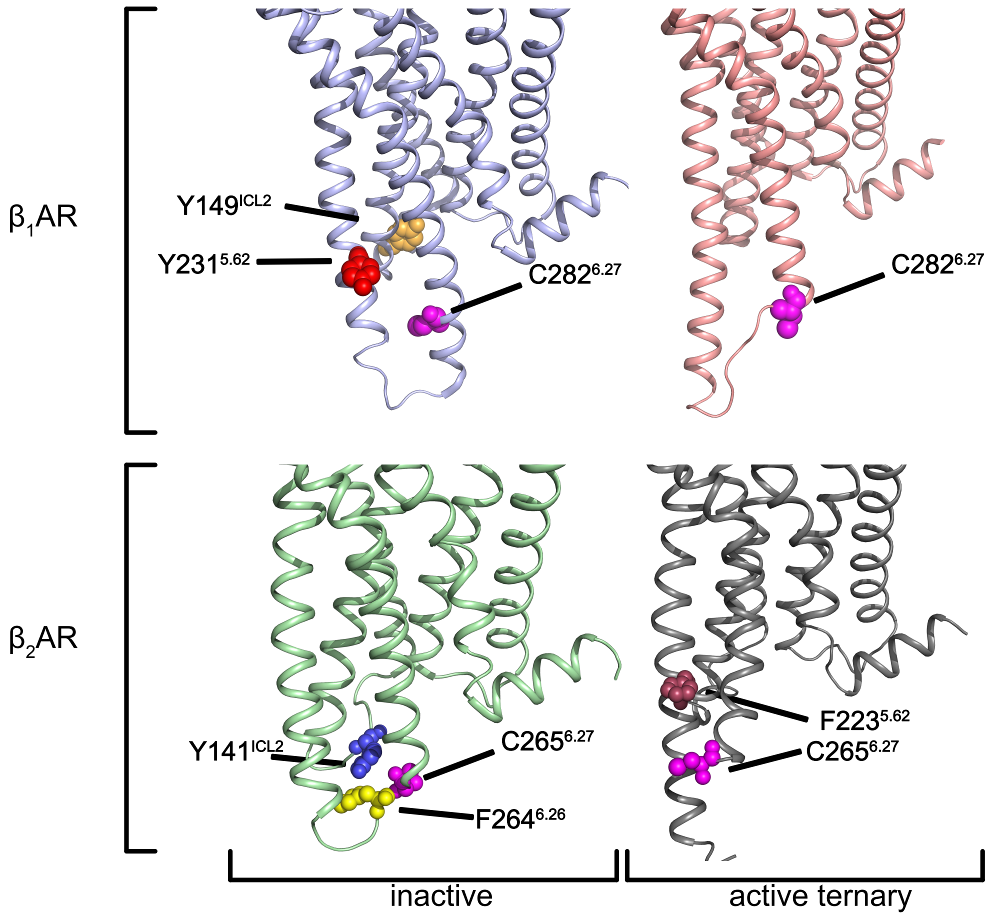

Supplementary Fig. 12 Comparing the location of aromatic residues in the vicinity of the TM6 reporter for β$_1$AR and β$_2$AR. The top panel shows the comparison of inactive (blue, PDB code 2YCY) and active (pink, PDB code 6H7J crystal structures of the β$_1$AR. The position of aromatic residues influencing the chemical shift of C282$^{6.27}$ (magenta) are shown in both structures as spheres. For the inactive β$_1$AR, Y149^{ICL2} and Y231$^{5.62}$ are shown in orange and red, respectively. Due to their extended distance to C282$^{6.27}$ they only minimally contribute to chemical shift changes. For the active β$_1$AR structure, there are no aromatic residues in the vicinity of C282$^{6.27}$. Between inactive and active β$_1$AR, therefore, the predicted changes in the aromatic environment of C282$^{6.27}$ are small, resulting in negligible chemical shift differences between both. This fits well with the experimental results. The bottom panel shows the same situation for inactive (green, PDB code 4GBR) and active (grey, PDB code 3SN6) β$_2$AR. Aromatic residues that have an effect on C265$^{6.27}$ (magenta) are Y141^{ICL2} and F264$^{6.26}$ in blue and yellow, respectively. Their close distance to C265$^{6.27}$ make them more likely to contribute towards chemical shift changes than for the corresponding position in the β$_1$AR, while the chemical shift of C265$^{6.27}$of β$_2$AR in the active state is influenced by the nearby F223$^{5.62}$ (purple). Between inactive and active β$_2$AR the changes in the aromatic environment of C265$^{6.27}$ are extensive therefore, resulting in substantial chemical shift changes due to variations in aromatic ring current shifts. Again, this is in agreement with the experimental data [2].

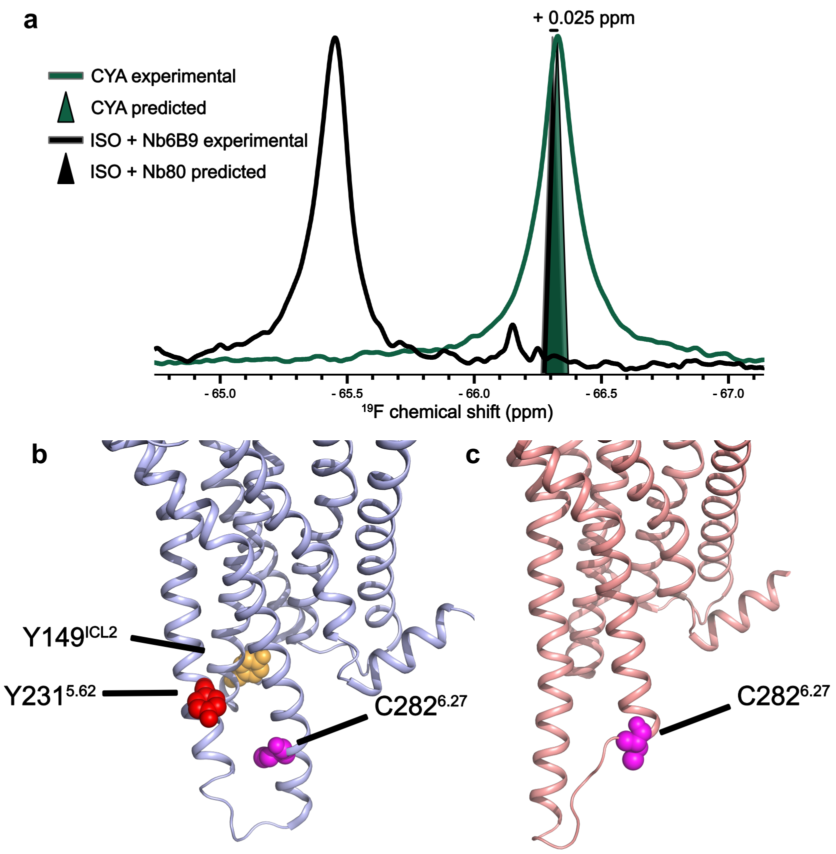

Supplementary Fig. 13 Comparison of experimentally observed and calculated chemical shifts for TM6 A282C$^{TET, 6.27}$. (a) Experimental ^{19}F NMR spectra are shown as solid lines for the cyanopindolol bound β_1AR (green) representing the inactive receptor and β_1AR in complex with full agonist isoprenaline and nanobody Nb6B9 (black) representing the signaling active receptor. Chemical shift predictions were performed using the molecular analysis software MOLMOL [3] (see Supplementary Table 3). Position of predicted chemical shifts are shown as large triangles for the cyanopindolol bound β_1AR (green) and full agonist isoprenaline and nanobody Nb80 bound β_1AR (black). The predicted position of the cyanopindolol bound receptor was normalized to the experimentally observed peak position of the cyanopindolol bound receptor. The distance between the predicted chemical shift positions of both receptor states is 0.025 ppm and is indicated as a black line. **(b)** The crystal structure of the cyanopindolol bound β_1AR (PDB code 2YCY) is shown as a blue cartoon. The position of A282C$^{TET, 6.27}$ is shown as magenta spheres. The only relatively close aromatic residues to A282C$^{TET, 6.27}$ are Y149^{ICL2} shown in orange and Y231$^{5.62}$ depicted in red. Both residues, however, are too distant to A282C^{TET} to considerably influence its chemical shift. **(c)** The crystal structure of the isoprenaline and nanobody Nb80 bound β_1AR (PDB code 6H7J) is shown as a pink cartoon. The position of A282C$^{TET, 6.27}$ is shown as magenta spheres. Based on the crystal structure coordinates MOLMOL [3] predicts no aromatic residues in the vicinity of A282C$^{TET, 6.27}$ that would contribute to a chemical shift change. Not surprisingly therefore inactive and pre-active states have very comparable chemical shifts.

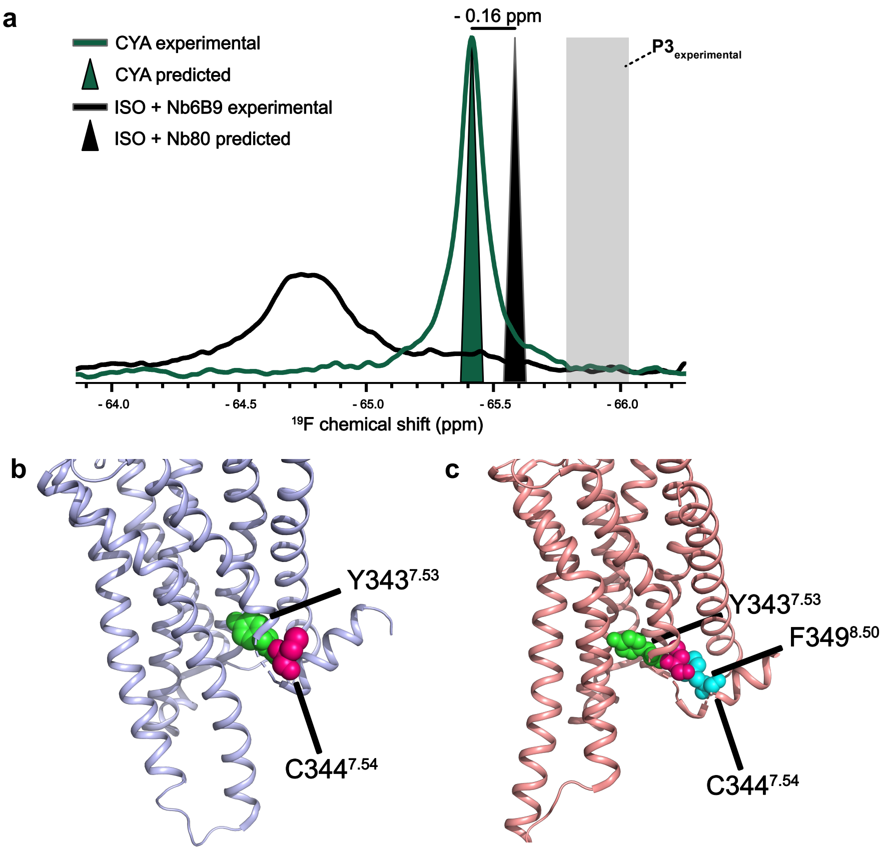

Supplementary Fig. 14 Comparison of experimentally observed and calculated chemical shift values for TM7 ^{TET}C344$^{7.54}$. (a) Experimental ^{19}F NMR spectra are shown as solid lines for the cyanopindolol bound β_1AR (green) for the receptor in a close to inactive conformation, and β_1AR in ternary complex with full agonist isoprenaline and nanobody Nb6B9 (black) for the signaling active receptor state. Predicted ^{19}F chemical shift changes were obtained in MOLMOL [3] using the Johnson-Bovey equation [4], considering solely contributions from aromatic ring current effects. In view of the general difficulty to predict ^{19}F chemical shifts [5,6], other less well understood contributions were excluded from the calculations. Hence, the predictions only reflect the effects of ring current shifts related to relative changes in position and orientation of aromatic residues that are in close vicinity to ^{TET}C344$^{7.54}$. The positions of the predicted chemical shifts are shown superimposed as filled triangle shapes for the cyanopindolol bound β_1AR (green) and for the full agonist isoprenaline bound and nanobody Nb80 coupled β_1AR (black). The predicted position of the cyanopindolol bound receptor was normalized to the experimentally observed peak position of the cyanopindolol bound receptor. The distance between the predicted chemical shift positions for the two receptor states is -0.16 ppm and is indicated as a black line. The experimental position of P3 is indicated with a grey box. The latter peak is found to be exchanging with P2. **(b)** The crystal structure of the cyanopindolol bound β_1AR (PDB ID 2YCY) is shown as blue cartoon. The position of ^{TET}C344$^{7.54}$ is shown as magenta spheres. Aromatic residues predicted to influence the chemical

shift of $^{TET}C344^{7.54}$ are shown. In this case this is the neighbouring $Y343^{7.53}$ depicted in green. β_1AR has a very low basal activity which would make the apo state the most representative for the inactive state of β_1AR. However, in the absence of an available crystal structure of the apo state the cyanopindolol structure was used instead. Weak agonist bound crystal structures of thermostabilized β_1AR are all very similar in the cytoplasmic region of TM6 and TM7, where they are believed to show a conformation representative of an inactive state [7]. **(c)** The crystal structure of the isoprenaline and nanobody Nb80 bound β_1AR (PDB ID 6H7J) is shown as pink cartoon. The position of $^{TET}C344^{7.54}$ is shown as magenta spheres. $Y343^{7.53}$ and $F349^{8.50}$ influence the predicted chemical shift of $^{TET}C344^{7.54}$ and are shown as green and cyan spheres, respectively. The orientation of the aromatic residues in the ternary state was assumed to be representative of their orientation in the pre-active state P3 (further details related to the calculations are provided in Supplementary Table 3).

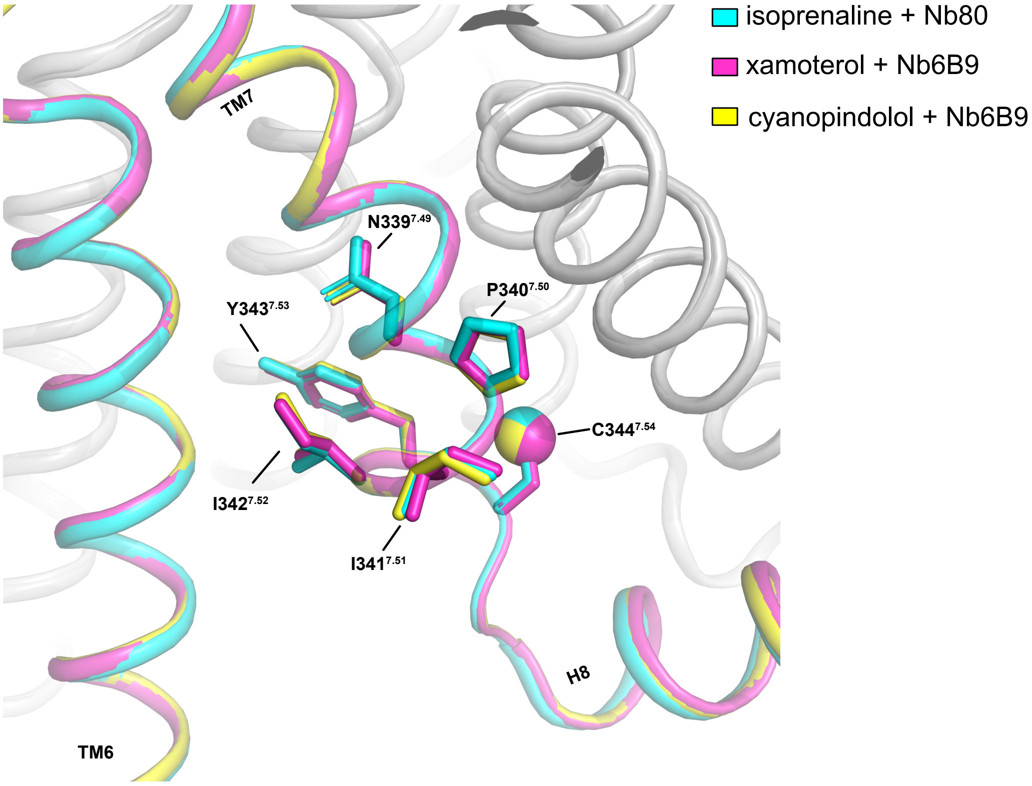

Supplementary Fig. 15 Overlay of active state crystal structures of β_1AR ternary complexes bound to agonists of varying efficacies and G_s mimicking nanobodies. The crystal structures of ternary complexes with cyanopindolol (yellow, PDB code 6H7O), xamoterol (pink, PDB code 6H7N) and isoprenaline (cyan, PDB code 6H7J). Enlarged side-on view of the cytoplasmic region of β_1AR TM7 and helix 8 with the residues of the conserved NpxxY$^{7.53}$ motif shown as sticks and the sulfur atom of the reporter C344$^{7.54}$ shown as a sphere. The overlay reveals the structures as virtually identical. The nanobody was excluded from the overlay for clarity.

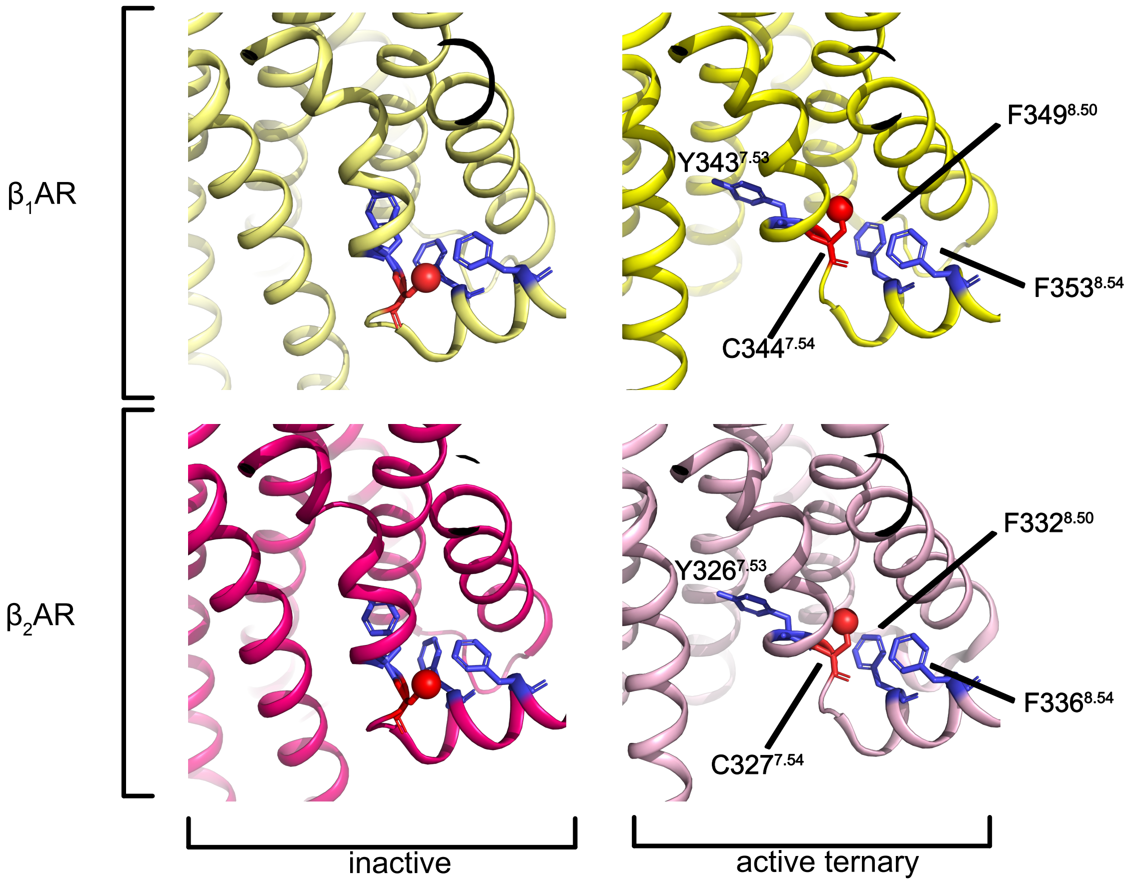

Supplementary Fig. 16 Comparing the location of aromatic residues in the vicinity of the TM7 reporter for β$_1$AR and β$_2$AR. The top panel shows the comparison of inactive (light yellow, PDB code 2YCY) and active (bright yellow, PDB code 6H7J) crystal structures of the β$_1$AR. The bottom panel shows the inactive (dark pink, PDB code 4GBR) and active (light purple, PDB code 3SN6) structures of β$_2$AR. The location of the TET probe is shown as red sphere attached to C344$^{7.54}$ or C327$^{7.54}$, respectively. Proximal aromatic residues are diplayed in blue. For both receptors, these residues correspond to the positions of Y$^{7.53}$, F$^{8.50}$, F$^{8.54}$. Both receptors show an inward rotation of TM7 upon activation which causes a change in the relative orientation of the C$^{7.54}$ probe to the aromatic residues.

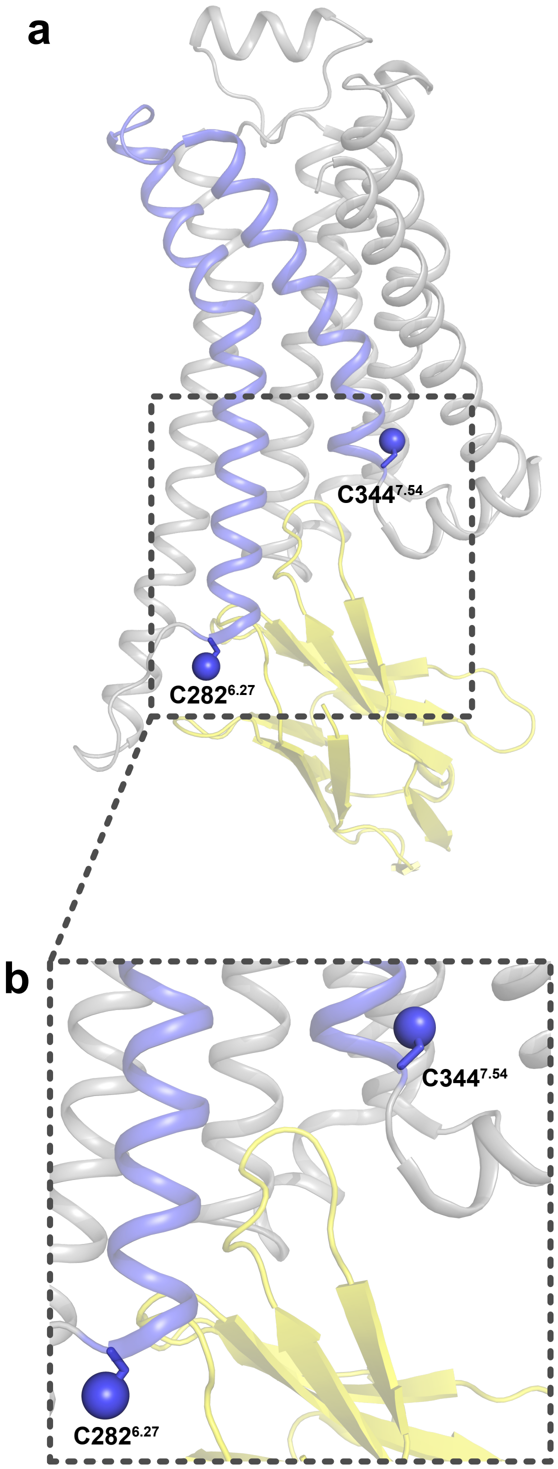

Supplementary Fig. 17 Ternary structure of the turkey β$_1$AR bound to the partial agonist xamoterol and the nanobody Nb6B9 showing the receptor in a conformation corresponding to the fully active state (PDB ID 6H7N). (a) Side-on view of the ternary complex with β$_1$AR shown in grey. TM6 and TM7 of the receptor are colored in blue with the TET labelling sites A282C$^{6.27}$ and C344$^{7.54}$ used in this study highlighted as blue spheres. The nanobody is colored in yellow. **(b)** Enlarged view of the cytoplasmic binding pocket.

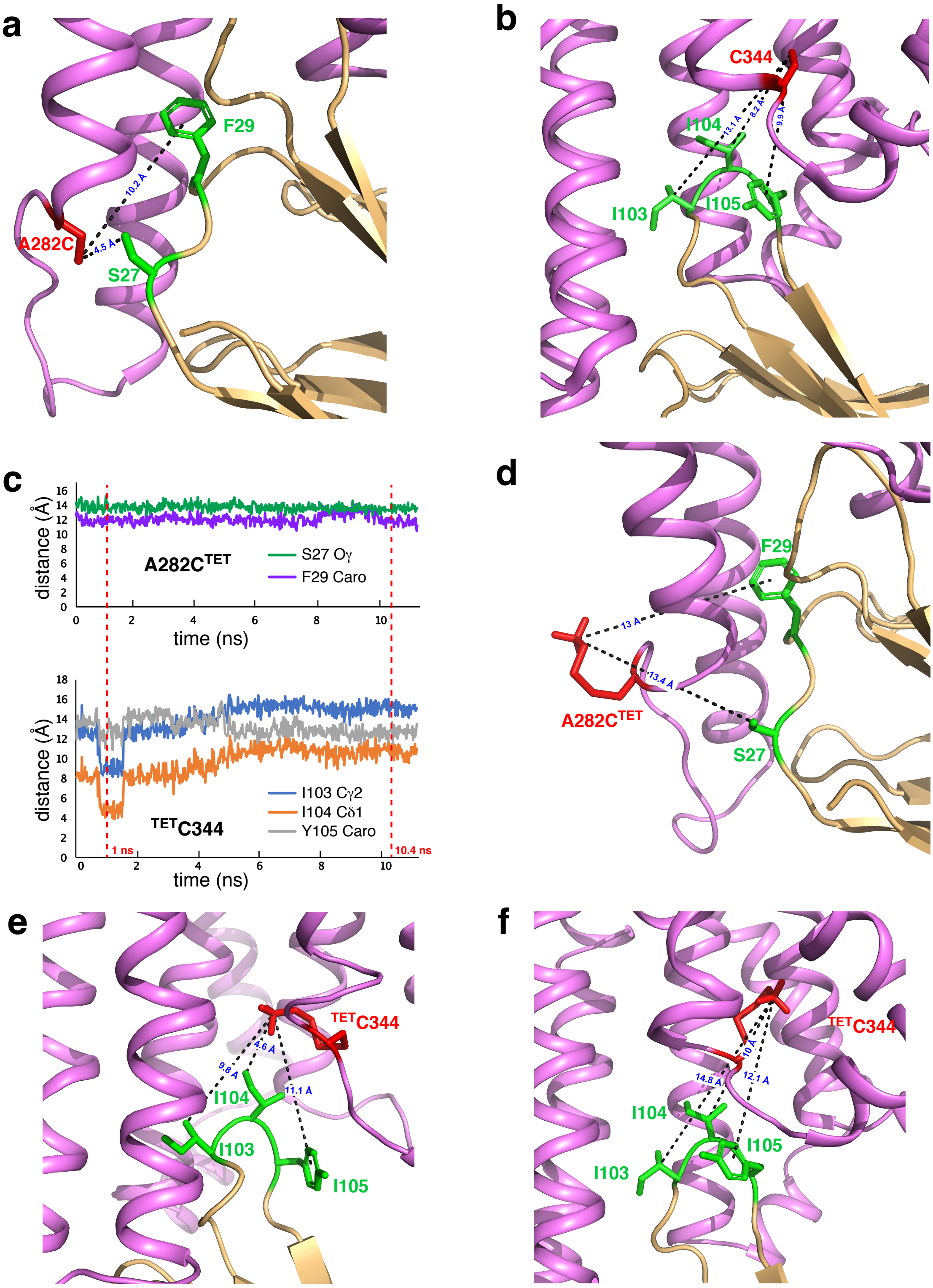

Supplementary Fig. 18 Nanobody residues in proximity to the ^{19}F NMR probes: receptor-nanobody binding interface in the ternary complex of xamoterol-bound turkey β_1AR coupled to nanobody Nb6B9 (PDB ID 6H7N). (a,b,d,e,f) Cartoon representation of the cytoplasmic region of β_1AR (pink) and the region of the nanobody (cream) interacting with the receptor. Nanobody residues in close proxmimity to the ^{19}F probe locations on TM6 and TM7 of β_1AR are represented with their side chains as sticks (green). The ^{19}F probe locations are approximated by: **(a)** Sγ of TM6 A282C$^{6.27}$ (red), **(b)** Sγ of TM7 C344$^{7.54}$ (red), **(d)** CF_3 of

A282C$^{TET,6.27}$ (red), and **(e, f)** CF_3 of $^{TET}C344^{7.54}$ (red). The shortest distances between nanobody and the ^{19}F probes are highlighted by dashed black lines, with the distances indicated in blue. Distances given in (a) and (c) are based on the unmodified coordinates of the crystal structure and correspond to a situation where the location of the ^{19}F probe is approximated by the Sγ position. Aiming to obtain better representative positions for the fluoro probes, TET tags were added to the Sγ positions resulting in A282C^{TET} and ^{TET}C344. The conformational energy of the structure with the tagged cysteines was subsequently minimized and a MD simulation conducted with the receptor embedded in a fully hydrated POPC bilayer. **(c)** MD trajectories showing the shortest distances between nanobody residues S27 (Oγ) or F29 (Caro) and the CF_3 of A282C^{TET} **(c, upper)**, and between the nanobody residues I103 (Cγ2), I104 (Cδ1) or Y105 (Caro) and the CF_3 of ^{TET}C344 **(c, lower)** as a function of the simulation time. For A282C^{TET} the distances remain very similar during the entire simulation. For ^{TET}C344, however, the simulation reveals two side chain orientations. A minor conformer at ca. 1 ns that exists for ca 8% of the simulation and shows shorter distances to the nanobody, while for the remainder of the simulation (92%) the distances are considerably larger. **(e)** Snapshot taken at 1 ns representative of the minor conformer (c, red dashed line) with the orientation of ^{TET}C344 pointing in direction of TM6 that results in reduced distances to the nanobody. **(f)** Snapshot taken at 10.4 ns (c, red dashed line) representative of the major conformer where the distances from the CF_3 of ^{TET}C344 to the nanobody are substantially increased.

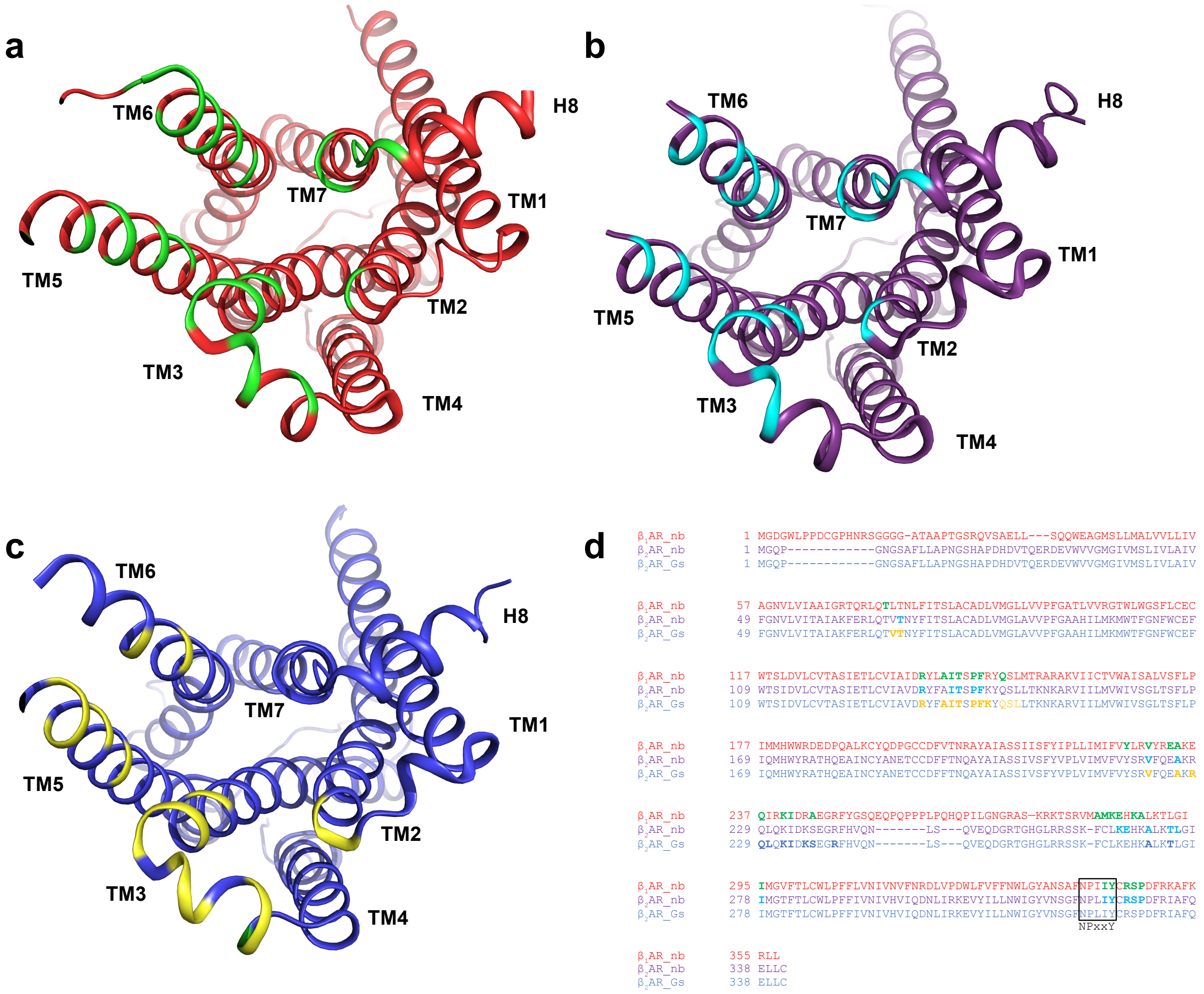

Supplementary Fig. 19 Binding interface receptor residues in ternary complex between β_1AR or β_2AR and intracellular binding partners (IBPs). Cytoplasmic view of β_1AR and β_2AR in ternary complex, with the residues with atoms within 3.8 Å of the binding partner highlighted.The IBPs are omitted from the figure for clarity. **(a)** The β_1AR (PDB code 6H7J) is shown as a red cartoon and residues in contact with the nanobody Nb80 are highlighted in green. The positions of the individual helices are indicated. **(b)** The β_2AR (PDB code 3P0G) is shown as purple cartoon and residues in contact with the nanobody Nb80 are highlighted in cyan. **(c)** The β_2AR (PDB code 3SN6) is shown as blue cartoon and residues in contact with the Gα subunit of the heterotrimeric G_s protein are highlighted in yellow. **(d)** Sequence alignment of the receptors shown in (a) to (c) with the IBP interacting receptor residues highlighted using the same colour scheme. The NPxxY$^{7.53}$ motif on TM7 is indicated by a box. While no TM7 interactions are observed in the G_s structure of β_2AR, interactions involving the TM7 region leading into H8 are similar for β_1AR and β_2AR bound to nanobodies, with five residues in the TM7-helix 8 region being part of the binding interface.

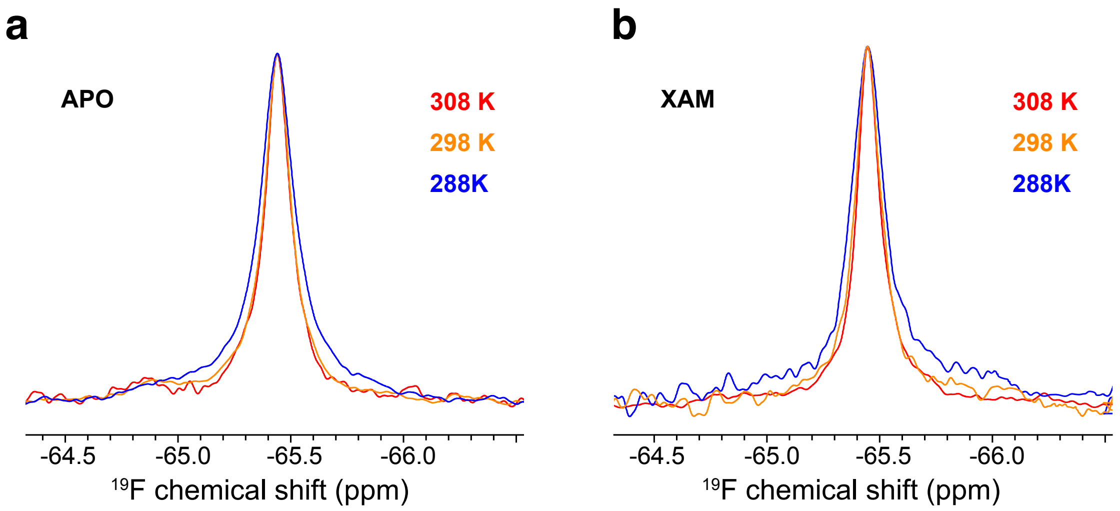

Supplementary Fig. 20 Temperature dependent ^{19}F NMR spectra of β_1AR TM7 ^{TET}C344$^{7.54}$ in the apo form and bound to xamoterol. Overlay of spectra showing the signal P2 recorded at the temperatures of 308 K (red), 298 K (orange) and 288 K (blue) for the receptor in the apo form **(a)** and bound to xamoterol **(b)**. Over the temperature range investigated there is no evidence of multiple signal components for the P2 signal.

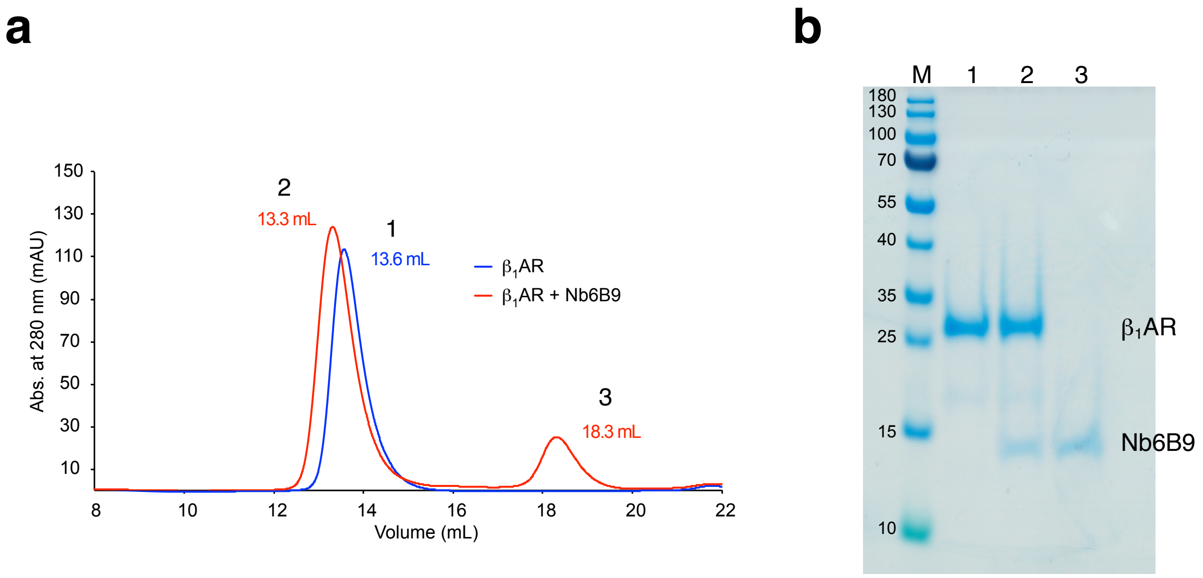

Supplementary Fig. 21 β_1AR ternary complex formation demonstrated by SEC and SDS-PAGE analysis. 14 μM β_1AR-m-CysΔC2 solubilized in LMNG were incubated with 1 mM isoprenaline and 21 μM Nb6B9. **(a)** SEC (Superdex S200 10/300) traces of isoprenaline-bound β_1AR-m-CysΔC2 in the absence (blue), and in the presence of nanobody Nb6B9 (red), showing formation of the ternary receptor complex (peak 2). **(b)** SDS-PAGE analysis of the SEC fractions. SEC fraction peak numbers and SDS-page lane numbers correspond to each other. M, molecular weight marker; lane 1, β_1AR; lane 2, ternary complex of isoprenaline-bound β_1AR-m-CysΔC2 complexed with Nb6B9 nanobody; lane 3, unbound nanobody.

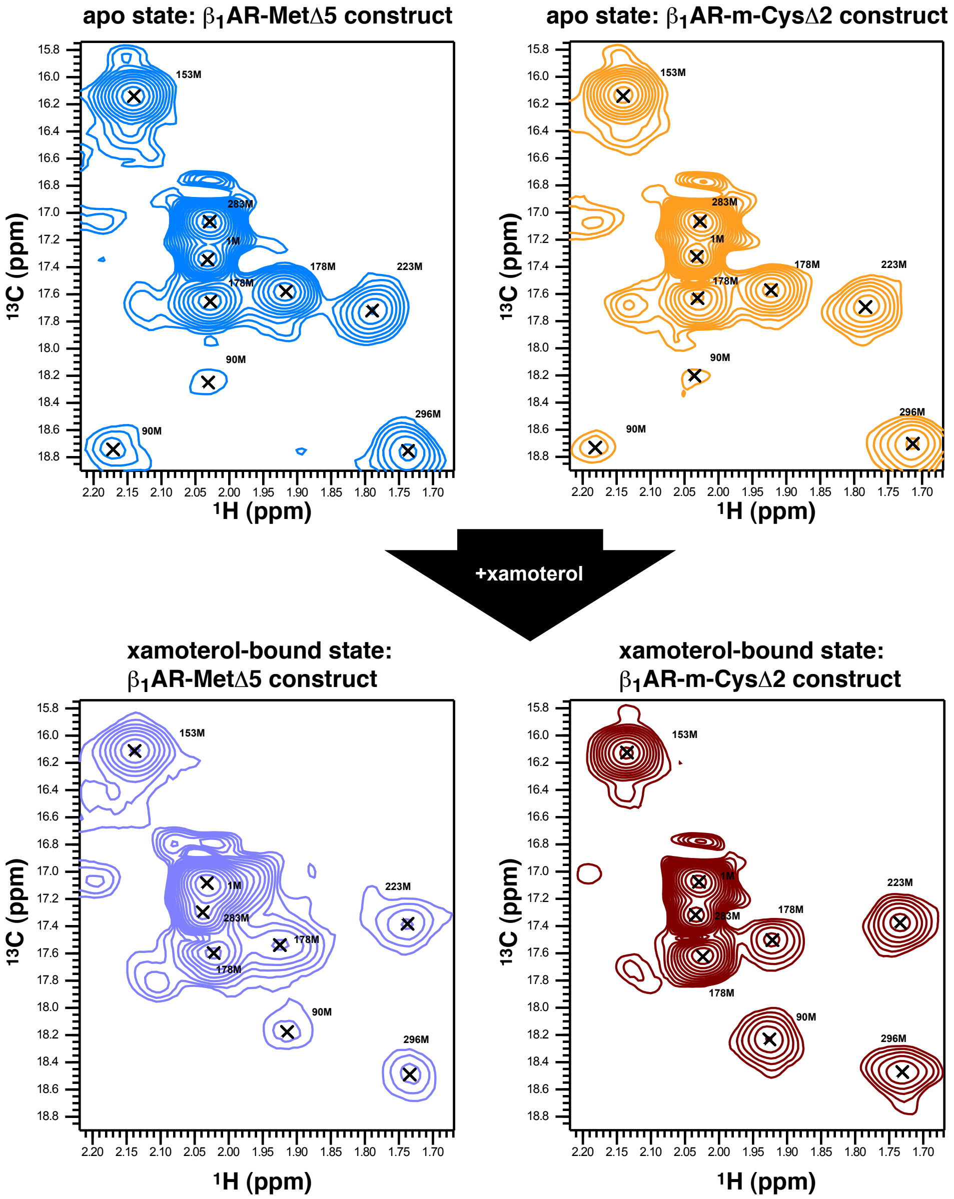

Supplementary Fig. 22 Receptor functionality of β_1AR-CysΔ2 demonstrated by ^{13}C methyl methionine NMR. Comparison of ^{1}H, ^{13}C HMQC spectra of ^{13}C-Met β_1AR-MetΔ5 (used in the study by Solt et al 2017 (ref [17] in main text) and ^{13}C-Met β_1AR-m-CysΔ2 with ^{TET}C344$^{7.54}$ (used in this study) in the apo form and bound to xamoterol. Both receptors show equal spectral appearance in the apo form and the same behaviour upon activation through binding of the partial agonist xamoterol. It is concluded that the two receptor constructs behave very similarly and that the presence of the CysΔ2 mutation and TET tagging at C344 has no deleterious effect on the functional response of the receptor construct β_1AR-m-CysΔ2. Experiments were recorded at 800 MHz (^{1}H) and 308 K.

Supplementary Table 1

Pharmacological properties of agonists used in this study

Ligand name	Log K_D[#]	G_S efficacy[$] (% isoprenaline)	Chemical structure (Head / Tail)
atenolol	-5.40 ± 0.06	2.1	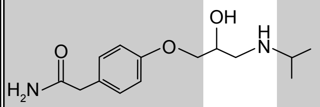
carvedilol	-9.43 ± 0.05	12	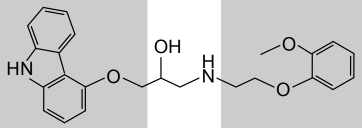
alprenolol	-7.96 ± 0.04	31	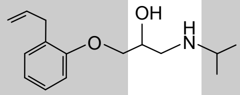
cyanopindolol	-10.89 ± 0.06	34	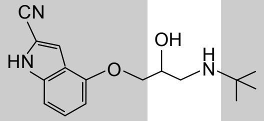
bucindolol	-9.43 ± 0.06	49	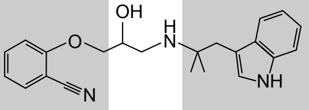
xamoterol	-6.58 ± 0.02	67	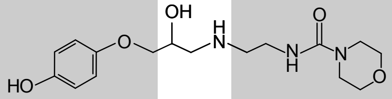
isoprenaline	-6.86 ± 0.08	100	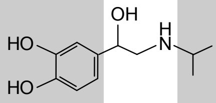
adrenaline	-6.01 ± 0.04	108	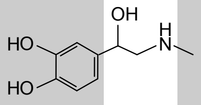

[#]Data taken from Baker et al. 2010 [8]

[$]Data taken from Baker et al. 2011 [9]

Supplementary Table 2

[19]F chemical shift positions and R_2 values of β_1AR TM6 A282C^{TET} and TM7 ^{TET}C344, for ligand-bound receptor and for receptor in ternary complex coupled to Nb6B9 (chemical shift positions and R_2 values were obtained from signal deconvolutions using Lorentzian lines)

				TM6 A282C^{TET}					TM7 ^{TET}C344							
				Chemical shifts§ (ppm)		R_2 (Hz)§			Chemical shifts§ (ppm)				R_2 (Hz)§			
			peak#	P1	P4	P1	P4	P1/P7‡	P2	P5	P2$	P6$	P2	P5	P2$	P6$
			state†	I_1	A^{G+}	I_1	A^{G+}	I/A^{G-}	I_1,I_2	A^{G+}	I_1,I_2	A^{G-}	I_1,I_2	A^{G+}	I_1,I_2	A^{G-}
**Ligand**	**Abbr.**	**Efficacy**	**Nb6B9£**	no	yes	no	yes	yes	no	yes	yes	yes	no	yes	yes	yes
ligand free	APO		2x	-66.33	-65.44	115	75	130	-65.42	-64.69	-65.42	-65.42	80	320	75	300
atenolol	ATE	2	-	-66.37		115			-65.43							
carvedilol	CVD	12	2x	-66.34	-65.45	118	75	120	-65.43	-64.57	-65.43	-65.55	80	250	82	250
alprenolol	ALP	31	-	-66.33		120			-65.41							
cyanopindolol	CYA	34	2x	-66.37	-65.47	122	95	120	-65.42	-64.71	-65.47	-65.55	80	230	80	330
Bucindolol	BUC	49	2x						-65.47	-64.68	-65.47	-65.50				
xamoterol	XAM	67	2x	-66.35	-65.44	130	92	170	-65.46	-64.78	-65.45	-65.51	100	270	103	240
xamoterol	XAM	67	5x						-65.46	-64.77	-65.46	-65.52	100	270	105	250
xamoterol	XAM	67	10x						-65.46	-64.78	-65.45	-65.52	100	260	100	260
isoprenaline	ISO	100	2x	-66.31	-65.45	140	106		-65.47	-64.77		(-65.42)	140	320		(200)
adrenaline	ADR	108	-						-65.47				160			

#Peak numbering as discussed in the main text and shown on the spectra
†Receptor states as discussed in the main text
§Chemical shift positions and R_2 values were obtained from deconvolution with Lorentzian lines
$For TM7 in the presence of nanobody the overlapped high field region containing P2 and P6 signals was deconvoluted with two Lorentzian lines (Supplementary Fig. 8) resulting in significantly improved fits for this region, as evidenced by the smaller residuals. For the isoprenaline complex the signal-to-noise of the P2/P6 region was too low to justify deconvolution using two Lorentzians lines. Instead, deconvolution of the P2/P6 region was conducted using a single Lorentzian line thus resulting in an R_2 value between the ones for the P2 and P6 signals.
‡The existence of P7 on TM6 is based on the observations for the corresponding peak P6 on TM7, which infers the presence of the (A^{G-}) form also for TM6. The chemical shift of P7 is almost identical to P1 and the presence of P7 therefore only shows via changes in linewidth as the amount of nanobody is varied. The existence of P7 is not discussed in the main text, but its presence mirrors the behaviour of P6 on TM7.
£The factors (2x, 5x, 10x) relate to the excess of Nb6B9 over β_1AR. 'no' means: in the absence of Nb6B9; 'yes' means: in the presence of Nb6B9

Supplementary Table 3
Predicted and observed ^{19}F chemical shift positions for β$_1$AR TM6 A282C and TM7 C344, for ligand-bound receptor and for receptor in ternary complex coupled to nanobody

		TM6 A282C					TM7 C344				
		Ring-current shifts		Experimental			Ring-current shifts		Experimental		
State	Mutation *	Predicted§	Δδ(CYA) ¥	Observed	Δδ(CYA, P1) ¥	Contributions > $\lvert 0.01 \rvert$¶	Predicted§	Δδ(CYA) ¥	Observed	Δδ(CYA, P2) ¥	Contributions > $\lvert 0.05 \rvert$¶
CYA#	L282C	-0.028	0	P1: -66.37	P1: 0	Tyr 149<br>Tyr 231	0.024	0	P2: -65.42	P2: 0	Tyr 343
ISO + Nb80$	A282C	-0.003	0.025	**+Nb6B9**<br>P4: -65.45	P4: +0.92	-	-0.133	-0.157	**+Nb6B9**<br>P5: -64.77<br>P3: -65.95	P5: +0.65<br><br>P3: -0.53	Tyr 343<br>Phe 349

#Predictions using PDB structure 2YCY, chain A
$Predictions using PDB structure 6H7J, chains A & C
*In order to predict the ring current shift for C282, crystal structures were mutated to cysteine using the mutagenesis function in Pymol [10], with the backbone-dependent rotamer giving minimum steric clashes selected
¶Residues contributing >|0.01| and > |0.05| ppm to the predicted ring-current shift for C282 and C344 respectively.
§Ring-current chemical shifts for cysteine Hγ were calculated based on the Johnson-Bovey equation [4] as implemented in MolMol [3] using the crystallographic coordinates. Protons were added using UCSF Chimera [11]. Calculations used the preferred crystallographic chain as reported in the GPCRdB [12].
¥Chemical shift difference calculated relative to the cyanopindolol-bound form. For experimentally observed shifts, the inactive state signal, P1 or P2 for C282 and C344 respectively, is used.

Supplementary Table 4
Primer sequences used for site-directed cysteine mutagenesis

**C85V**	Forward	GTGGCCGACCTGGTGAT
	Reverse	GGCCAGCGAGGTGATGAAG
**C163L**	Forward	CTGACCGTCTGGGCCATCTCC
	Reverse	GATGATGACCTTGGCCCGAGC
**C344S**	Forward	ATCATCTACTCCCGCAGCCC
	Reverse	GGGGTTGGCAGCAGAGTT
**A282C**	Forward	TGTCGCCTGCATGAGGGAACACAAA
	Reverse	CGGGACGTCTTCCTCTTGCTA

Supplementary Note 1: β_1AR constructs and ^{19}F labelling of cysteines

Previously we studied the activation of the β_1AR using ^{13}C methyl-methionine NMR spectroscopy, focusing on the TM region of the receptor [13]. To relate this ^{19}F NMR study to our previous investigation, we set out from the same β_1AR-MetΔ5 construct [13], named β_1AR-m, that relative to wild type contains three thermostabilising mutations, with the number of methionine residues reduced by five (Supplementary Fig. 1) (see Methods). The functionality of the construct was shown previously [13]. Of the six reduced cysteines available in β_1AR-m, native C344$^{7.54}$ was suitable for fluoro-tagging to report on the cytoplasmic region of TM7 during receptor activation (Supplementary Fig. 1a). A separate reporter cysteine at the end of TM6 was introduced in a second construct as A282C$^{6.27}$, while removing the TM7 cysteine via a C344S$^{7.54}$ mutation, resulting in β_1AR-m-TM6 (Supplementary Fig. 1b). To permit a direct comparison with our previous ^{13}C NMR study all experiments were conducted with receptor solubilized in LMNG micelles.

Tests using 3-bromo-1,1,1-trifluoroacetone (BTFA) resulted in the labelling of multiple cysteine sites and were abandoned, despite the use of constructs where two accessible Cys residues on TM2 and TM4 were replaced as C85V$^{2.48}$ and C163L$^{4.47}$, leading to β_1AR-m-CysΔ2 (Supplementary Fig. 1a) and β_1AR-m-TM6-CysΔ2 (Supplementary Fig. 1b). Further attempts with 2-bromo-N-[4-(trifluoromethyl)phenyl]acetamide (BTFMA) resulted in receptor that was unable to bind nanobody Nb6B9 (Supplementary Fig. 3). Labelling with 2,2,2-trifluoroethanethiol (TET) following the described two step reaction (Supplementary Fig. 2) [1], resulted in the required selective ^{19}F tagging of β_1AR-m-CysΔ2 at ^{TET}C344$^{7.54}$ and β_1AR-m-TM6-CysΔ2 at A282C$^{TET,6.27}$ (Supplementary Fig. 4a). ^{19}F labelled CysΔ2 receptor constructs showed identical responses towards ligand and nanobody binding as the β_1AR-m based receptor constructs, but the presence of C85$^{2.48}$ and C163$^{4.47}$ in the latter produced unstable receptor due to tagging at multiple internal sites, which substantially reduced the yield of receptor labelled exclusively at A282C$^{6.27}$ or C344$^{7.54}$, respectively (Supplementary Fig. 5). Hence, all NMR studies were conducted using the two CysΔ2 constructs of β_1AR. The ^{19}F labelled NMR samples were purified to homogeneity (Supplementary Fig. 4b) and ^{19}F NMR assignments were obtained based on the unique complementarity of the non-overlapping A282C$^{TET, 6.27}$ and ^{TET}C344$^{7.54}$ signals (Supplementary Fig. 4a). While all of the receptors were stable over several days at 308 K the slow cleavage of the S-S bond resulted in the gradual release of small amounts of free TET over time, which became visible in the NMR spectra. This however does not interfere with any of our investigations.

Supplementary Note 2: Solvent accessibility of the TM7 ^{TET}C344 environment

The response of the TM7 $^{TET}C344^{7.54}$ environment upon addition of a water-soluble Gd^{3+} paramagnetic relaxation reagent was investigated for xamoterol-bound β_1AR-m-CysΔ2 in the presence of a two-fold excess of nanobody through titration with increasing amounts (0 - 5 mM) of gadopentetic dimeglumine (Magnevist). The reaction conditions permitted a side-by-side comparison of the ligand-bound receptor via the P2 signal and the ternary receptor via the P5 signal. Signal intensity losses for P5 related to Gd^{3+} addition were more pronounced than for P2 and increases in R_2 values (determined from a two-point measurement) for P5 were more substantial than for P2 (Supplementary Fig. 11). Relaxation enhancements $\varepsilon = 32.87\ s^{-1}mM^{-1}$ for P5 and $\varepsilon = 8.29\ s^{-1}mM^{-1}$ for P2 were obtained from the slopes of the linear fits to the R_2 rates. The measurement series established the ternary $^{TET}C344^{7.54}$ receptor environment relating to the P5 signal to be significantly more solvent-accessible than in the ligand-bound receptor as assessed by the P2 signal.

Supplementary Note 3: Response of TM6 A282C$^{TET, 6.27}$ to agonist binding

The P1 signal for A282C$^{TET,6.27}$ showed only a relatively small response to ligand binding upon probing the receptor with a range of agonists varying in efficacy (Fig. 2a, Supplementary Fig. 6a). A small amount of exchange broadening to R_2 became apparent when comparing the apo receptor with isoprenaline bound β_1AR, indicating the presence of smaller amounts of μs-to-ms conformational dynamics when bound to higher efficacy ligands (Supplementary Table 2). Compared to our previous methionine ^{13}C NMR study, this relatively small effect initially appeared counterintuitive, as β_1AR-m showed strong responses in ^{1}H and ^{13}C to agonist activation as evidenced by a range of reporters, including those on TM4, TM5 and TM6 (M153$^{4.37}$, M178$^{4.62}$, M223$^{5.54}$ and M296$^{6.41}$) [13]. Also, as shown by Liu et al. for β_2AR, the equivalent position, $^{TET}C265^{6.27}$, on TM6 was observed in a slow-exchanging equilibrium between two states postulated as inactive and active receptor forms, that changed their populations in an agonist-efficacy dependent manner; in contrast to our observations with P1 [2]. It has been shown that aromatic residues in the proximity of a fluoro probe can have a considerable effect on the position of the observed ^{19}F NMR signal via ring current shifts [14]. Inspection of the molecular environment of A282C$^{6.27}$ in β_1AR showed a complete lack of aromatic residues in its vicinity, in contrast to the equivalent region in β_2AR (Supplementary Fig. 12) or to M296$^{6.41}$ in β_1AR [13]. Using the β_1AR crystal coordinates of several ligand-bound structures we calculated the expected ring current shifts for A282C^{TET} for different states compared to the inactive state (PDB code 2YCY) (Supplementary Fig. 13, Supplementary Table 3). In all cases, these calculated shift changes were smaller than 0.05

ppm, even when considering β_1AR active receptor states in ternary complex with nanobody, emphasizing that due to the local environment lacking aromatic residues and A282C$^{6.27}$ pointing towards the solvent, in the absence of changes in solvent accessibility the fluoro probe is unable to report on conformational changes at this position. Unsurprisingly therefore, the TM6 signal showed relatively minor changes upon ligand binding to the receptor (Supplementary Fig. 6a). It can be assumed therefore that the small amount of additional broadening observed in the isoprenaline bound form is related to exchange between conformations that differ only little in their chemical environments. Little chemical shift response in this region was also observed in the study by Isogai et al. for the backbone amide environment of V280$^{6.25}$, consistent with our data [15]. As evidenced in our study by the increase in R_2 values for P1 from the apo receptor to the isoprenaline bound form (Supplementary Table 2), it can be assumed that despite the lack of chemical shift perturbations, conformational interchange on TM6 is taking place. However, the similarity in chemical shifts between exchanging states when compared to β_2AR places this process in β_1AR on the fast-to-intermediate timescale, with relatively minor impact on the observed linewidth of P1.

Supplementary Note 4: Increased solvent accessibility of the TM7 ^{TET}C344 environment in the ternary complex

Titration with the paramagnetic relaxation reagent Magnevist established the cytoplasmic TM7 environment of the ternary complex to be more solvent accessible than in the ligand bound states. For TM7 C344$^{7.54}$ this supports the proposition where the observed downfield shift of P5 (compared to P2, P3) is caused by a greater solvent exposure in the ternary complex as the ^{19}F probe relocates from the more hydrophobic environment sampled in the ligand-bound states (Fig. 5). Based on this accessibility data obtained with xamoterol as ligand it seems reasonable to suggest that the comparable chemical shift changes observed upon formation of the ternary complexes with the other agonists relate to similar effects. Further, as the size of the chemical shift changes between ligand-bound receptor and ternary complexes for TM6 and TM7 are of similar size we suggest that a similar explanation holds also for the changes observed for A282C$^{TET,\ 6.27}$ on TM6. Other potential sources contributing towards the observed chemical shift changes need to be considered, such as the proximity of nanobody residues in or near the binding interface in the ternary complexes (Supplementary Fig. 17). We assessed the likely contribution of such effects by measuring the distances of nanobody residue side chains to the Sγ positions of A282C and C344, respectively, using the crystal structure of β_1AR in the ternary state in complex with xamoterol and Nb6B9 (PDB ID 6H7N) (Supplementary Fig. 18a,b). However, the Sγ locations only provide an approximate estimate of the ^{19}F probe positions. To obtain more representative distances we added the TET moieties onto the aforementioned cysteine residues and conducted a 12 ns MD

simulation of the ternary complex equilibrated in a fully hydrated POPC lipid bilayer (Supplementary Fig. 18c). While the simple analysis using distances to Sγ suggested S27 as a residue close to A282C (4.5 Å) (Supplementary Fig. 18a), the simulation showed no proximal residues within 13 Å of the A282C^{TET} CF_3 group (Supplementary Fig. 18d). For C344 all distances to Sγ were relatively large, with I104 closest at 8.2 Å (Supplementary Fig. 18b). The simulation revealed a minor conformer with a population of only 8% with a short distance from the CF_3 of ^{TET}C344 to I104 Cδ1(4.6 Å) (Supplementary Fig. 18e), while in contrast for the remaining time of the simulation (92% population) all distances in the sampled conformers were beyond 8 Å (Supplementary Fig. 18f).

Based on these distances we did not find any conclusive evidence for nanobody residue proximity related effects as a substantial contribution towards the observed changes in ^{19}F chemical shifts. Overall, the increased solvent-accessibility for TM6 A282C and TM7 C344 following a move into a less hydrophobic environment seems to provide the most consistent explanation for the prominent downfield shifts observed upon ternary complex formation, as proposed above.

Supplementary Note 5: Interactions between β_1AR and Nb6B9 in the ternary complex

A caveat needs to be added regarding the usage of nanobodies such as Nb6B9 as their primary purpose resides in stabilizing a GPCR in a native-like fully active state, while they are a poor mimetic of extended Gα_s function. The similarity of nanobody as well as G_s bound structures of β_2AR in the TM7-helix 8 region is very high. However, in the crystal structure of β_2AR in the full G_s protein complex TM7 lacks direct interactions with the heterotrimeric G_s protein, while both β_2AR and β_1AR in nanobody complexes show five residues in the TM7-helix 8 region that form contacts, including the last two residues of the NPxxY$^{7.53}$ motif (Supplementary Fig. 19) [16]. Crystallography shows several ternary Nb6B9 and Nb80 complexes of β_1AR bound to a range of agonists of varying efficacy with TM7 conformations near the NPxxY motif that are nearly identical (Supplementary Fig. 15). This is in clear contrast to our NMR work in solution. Possibly the differences are the result of crystal packing effects making the static structure information in this area of the receptor unreliable.

Supplementary Methods: BTFA/BTFMA ^{19}F labelling protocol

Purified β_1AR receptor eluted from an NiNTA purification was collected and the solution adjusted to 10 μM in protein via concentration (Amicon Ultra-15 spin concentrator with 50 kDa molecular weight cutoff). The labelling reagent 3-bromo-1,1,1-trifuluoroacetone (BTFA) or 2-bromo-N-(4-(trifluoromethyl)phenyl)acetamide (BTFMA), respectively, was added in a tenfold molar excess to a cold solution of the receptor and stirred gently for 1 h at 4 ˚C. The labelling reaction was terminated through removal of the labelling reagent through dilution concentration (1000x) or desalting using a HiTrap desalting column (GE Healthcare) into buffer containing 50 mM Tris pH 8, 350 mM NaCl and 0.02% (w/v) LMNG. The labelled receptor was further purified by alprenolol ligand affinity chromatography and eluted with either 1 mM atenolol or 0.1 mM alprenolol.

Supplementary References

1. Klein-Seetharaman, J., Getmanova, E. V., Loewen, M. C., Reeves, P. J. & Khorana, H. G. NMR spectroscopy in studies of light-induced structural changes in mammalian rhodopsin: applicability of solution (19)F NMR. *Proc. Natl. Acad. Sci. U. S. A.* **96**, 13744–13749 (1999).
2. Liu, J. J., Horst, R., Katritch, V., Stevens, R. C. & Wüthrich, K. Biased signaling pathways in β_2-adrenergic receptor characterised by ^{19}F-NMR. *Science* **335**, 1106–1110 (2012).
3. Koradi, R., Billeter, M. & Wüthrich, K. MOLMOL: A program for display and analysis of macromolecular structures. *J. Mol. Graph.* **14**, 51–55 (1996).
4. Johnson, C. E. & Bovey, F. A. Calculation of nuclear magnetic resonance spectra of aromatic hydrocarbons. *J. Chem. Phys.* **29**, 1012–1014 (1958).
5. Kitevski-LeBlanc, J. L. & Prosser, R. S. Current applications of ^{19}F NMR to studies of protein structure and dynamics. *Prog. Nucl. Magn. Reson. Spectrosc.* **62**, 1–33 (2012).
6. Ye, L. *et al.* Mechanistic insights into allosteric regulation of the A_{2A} adenosine G protein-coupled receptor by physiological cations. *Nat. Commun.* **9**, 1372 (2018).
7. Warne, T. *et al.* Structure of a β1-adrenergic G-protein-coupled receptor. *Nature* **454**, 486–491 (2008).
8. Baker, J. G. A full pharmacological analysis of the three Turkey β-adrenoceptors and comparison with the human β-adrenoceptors. *PLoS One* **5**, (2010).
9. Baker, J. G., Proudman, R. G. W. & Tate, C. G. The pharmacological effects of the thermostabilising (m23) mutations and intra and extracellular (β36) deletions essential for crystallisation of the turkey β-adrenoceptor. *Naunyn. Schmiedebergs. Arch. Pharmacol.* **384**, 71–91 (2011).
10. The PyMOL Molecular Graphics System Version 2.0.
11. Pettersen, E. F. *et al.* UCSF Chimera - A visualization system for exploratory research and analysis. *J. Comput. Chem.* **25**, 1605–1612 (2004).
12. Pándy-Szekeres, G. *et al.* GPCRdb in 2018: adding GPCR structure models and ligands. *Nucleic Acids Res.* **46**, D440–D446 (2018).
13. Solt, A. S. *et al.* Insight into partial agonism by observing multiple equilibria for ligand-bound and Gs-mimetic nanobody-bound β1-Adrenergic receptor. *Nat. Commun.* **8**, 1–12 (2017).
14. Liu, D. & Wüthrich, K. Ring current shifts in ^{19}F-NMR of membrane proteins. *J. Biomol. NMR* (2016). doi:10.1007/s10858-016-0022-4
15. Isogai, S. *et al.* Backbone NMR reveals allosteric signal transduction networks in the β 1-adrenergic receptor. *Nature* **530**, 237–241 (2016).
16. García-Nafría, J. & Tate, C. G. Cryo-EM structures of GPCRs coupled to Gs, Gi and Go. *Mol. Cell. Endocrinol.* **488**, 1–13 (2019).
